# Chaperone Requirements for Biosynthesis of the Trypanosome Variant Surface Glycoprotein

**DOI:** 10.1371/journal.pone.0008468

**Published:** 2010-01-05

**Authors:** Mark C. Field, Tatiana Sergeenko, Ya-Nan Wang, Susanne Böhm, Mark Carrington

**Affiliations:** 1 Department of Pathology, University of Cambridge, Cambridge, United Kingdom; 2 College of Veterinary Medicine, China Agricultural University, Beijing, People's Republic of China; 3 Department of Biochemistry, University of Cambridge, Cambridge, United Kingdom; BMSI-A*STAR, Singapore

## Abstract

**Background:**

*Trypanosoma brucei* does not respond transcriptionally to several endoplasmic reticulum (ER) stress conditions, including tunicamycin or dithiothreitol, indicating the absence of a conventional unfolded protein response. This suggests divergent mechanisms for quality control (QC) of ER protein folding and export may be present in trypanosomes. As the variant surface glycoprotein (VSG) represents ∼90% of trypanosome plasma membrane protein, it is possible that VSG has evolved to fold efficiently to minimize ER folding burden.

**Methodology/Principal Findings:**

We demonstrate the presence of a QC system by pharmacological inhibition of the trypanosome 26S proteasome. This indicates active proteasome-mediated VSG turnover as ∼2.5 fold more VSG is recovered from cell lysates following MG132 inhibition. An *in silico* scan of the trypanosome genome identified 28 open reading frames likely to encode polypeptides participating in ER nascent chain maturation. By RNA interference we monitored the importance of these gene products to proliferation, VSG abundance and cell morphology. 68% of the cohort were required for normal proliferation, and depletion of most of these factors resulted in increased VSG abundance, suggesting involvement in ERQC and degradation.

**Conclusions/Significance:**

The retention of genes for, and the involvement of many gene products in, VSG folding indicates a substantial complexity within the pathways required to perform this role. Counterintuitively, for a super-abundant antigen VSG is apparently made in excess. The biosynthetic excess VSG appears to be turned over efficiently by the proteasome, implying that considerable VSG is rejected by the trypanosome ERQC mechanism. Accordingly, the VSG polypeptide is not well optimized for folding, as only ∼30% attains the native state. Finally as much of the core ERQC system is functionally conserved in trypanosomes, the pathway has an ancient evolutionary origin, and was present in the last common eukaryotic ancestor.

## Introduction

In higher eukaryotic cells ∼20% of proteins are targeted to the endoplasmic reticulum (ER) to populate endomembrane compartments or for secretion [1], [2]. This represents a considerable burden to the ER in terms of overall molecular flux and in providing a suitable environment for folding nascent chains and assembling multi-subunit complexes. The ER lumen has a high Ca^2+^ concentration and is oxidizing, reflected in the abundance of Ca^2+^-dependent chaperones and protein disulphide isomerases (PDIs) that assist polypeptide folding following translocation into the ER *via* Sec61 [3]. Many examples of proteins failing to fold efficiently are known, and several are associated with pathology. Classic examples are CFTR^ΔF504^ and various α1-antitrypsin variants [4], [5]. Decreased folding competence of these allelic variants reduces activity, resulting in cystic fibrosis or emphysema, respectively. Further, accumulation of misfolded protein aggregates may result in amyloid-related disease [6].

**Figure 1:**
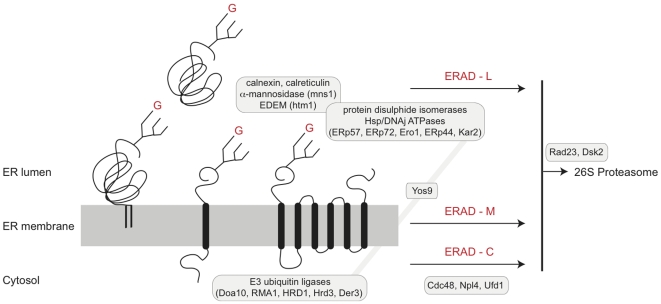
ER-associated degradation pathways. A schematic view of ERAD is shown, emphasizing the pathways acting on lumenal (ERAD-L), membrane (ERAD-M) and cytosolic (ERAD-C) portions of ER proteins. The ER membrane is shown as a gray rectangle into which are embedded a GPI-anchored glycoprotein (left), a single pass *trans*-membrane glycoprotein (center) and a polytopic membrane glycoprotein (right). *Trans*-membrane domains are represented as black lozenges, and Glc_1_Man_9_GlcNAc_2_ N-glycans by a tri-branched icon; red G = glucose. A lumenal glycoprotein is shown at top. Cohorts of gene products involved in various stages of folding, quality control, extraction and proteasome delivery are shown in boxes. All pathways require participation of Cdc28, while lumenal factors responsible for folding and quality control participate only in ERAD-L/M. Extraction requires ubiquitylation, and is mediated by RING-containing E3 ubiquitin ligases, including Doa10 and the HRD complex. Diagram is based on Hirsch *et al.*, [3], with simplifications. The present study was restricted to ERAD-L factors, together with trypanosome Yos9. Critically, Yos9 interacts with both lumenal components (Kar2) and cytoplasmic E3 ligases.

Cellular mechanisms for disposal of malfolded proteins and response to rapid increases in non-native polypeptide abundance within the ER are well defined in mammalian cells and *Saccharomyces cerevisiae* [3], [6], [7]. Folding within the ER, mediated by chaperones, PDIs and cycles of glucosylation/deglucosylation leads to either successful completion and export or, retrotranslocation into the cytosol. The general features of early steps of N-glycosylation and the glucosylation cycle are highly conserved amongst higher eukaryotes (Figure 1). There are three ER-associated degradation (ERAD) pathways; lumenal (L), membrane (M) and cytosolic (C) [3], [8]. Retrotranslocation is accompanied by ubiquitylation and targeting to the cytoplasmic proteosome for degradation. All three paths involve a complex consisting of Cdc48 (an AAA ATPase), Ufd1 and Npl4, for extraction of malfolded proteins from the ER [3], [9].

The ERAD-L pathway is the best characterized and includes BiP, PDIs, calnexin/calreticulin and a group of mannose-binding proteins (EDEMs) recognizing processed oligomannosidic N-glycans. Rejection of a malfolded polypeptide requires mannosidase II action to produce a Man_8_GlcNAc_2_ oligosaccharide, which is recognized by EDEM [10]. ERAD-L also requires Yos9, a mannose-binding lectin that regulates retrotranslocation and interacts with BiP and Hrd3. For ERAD-M, lumenal domains are subjected to a similar quality control and folding pathway as soluble proteins, while ERAD-C requires additional participation of cytosolic Hsp70 and Hsp40 chaperones [3]. Increased levels of unfolded proteins are sensed by IRE1, a *trans*-membrane ER kinase [11]. After stimulation IRE1 is cleaved to produce a splicing factor XBP1 and ultimately results in increased transcription of ERAD and unfolded protein response (UPR) genes. A second ER membrane kinase, PERK also mediates increased ERAD/UPR gene transcription, while a third path is mediated by ATF6α. All three factors are ordinarily bound by BiP; increased folding burden is believed to result in BiP releasing IRE1, PERK and ATF6α [12].

Post-ER trafficking is mediated by inclusion of correctly folded polypeptides into vesicles at the transitional ER (tER) [13]. At least two distinct pathways are present as vesicle populations enriched in glycosylphosphatidylinositol (GPI) or TMD cargo can be isolated, suggesting selection *via* the membrane anchor [14]–[17]. Supporting a model for selective anteriograde transport based on membrane association is the more profound importance to GPI-anchored than TMD protein transport of Lag1p and CERT, which participate in glycolipid synthesis [18], [19].

*Trypanosoma brucei*, the African trypanosome, is highly divergent from yeast and mammals, but the general features of the trypanosome endomembrane system are conserved [20], [21]. Little is known concerning protein folding factors but it has been reported that trypanosomes lacks a UPR [22], [23]. Further, IRE1 and ATF6α are absent from *T. brucei* and the PERK ortholog is located at the flagellar pocket, suggesting divergent function [24]. The trypanosome surface is dominated by the ∼58kDa variant surface glycoprotein (VSG), a predominantly α-helical, homodimeric GPI-anchored antigen expressed at 1×10^7^ copies, roughly 90% of cell surface protein [25]. VSG is highly stable with a T_1/2_ exceeding 72 hours, corresponding to over ten cell cycles [26]. VSG trafficking to the surface is normally rapid [27], [28] and disruption of secondary structure leads to decreased surface expression [29], [30]. N-glycosylation is required for stable surface expression while preventing GPI-anchor addition leads to ER retention [30]. These features suggest that despite the absence of the UPR, trypanosomes likely possess ERAD pathways, but the contributions of trypanosome chaperones are undefined [20]. Significantly, ongoing VSG biosynthesis is essential as suppression leads to growth arrest [31]. Trypanosomatids lack the Glc_3_Man_9_GlcNAc_2_ structure, but do transfer a Glc_1_Man_9_GlcNAc_2_ oligosaccharide to nascent chains [32]. Glc_1_Man_9_GlcNAc_2_ is capable of transient deglucosylation/reglucosylation [20], [33]. Interestingly, *T. brucei* encodes a calreticulin orthologue, but not calnexin, suggesting simpler glucosylation quality control (QC) is sufficient (Engster *et al.*, 2007). It is unknown if there is any selectivity in the generation of ER-derived transport vesicles but a Lag1 ortholog, multiple copies of p23/24 and three Rab proteins, Rab1A, Rab1B and Rab2, likely mediating ER to Golgi transport are present [34].

The extreme level of VSG expression may suggest that trypanosomes evolved for efficient biosynthesis of this particular molecule or that VSG is intrinsically efficient at folding and export. In support of this is the observation that additional surface molecules adopt a VSG-related fold, including the serum resistance-associated gene product (SRA), the transferrin receptor (ESAG6/7) and two families of invariant surface glycoproteins, ISG65 and ISG75 [35], [36]. In contrast to VSG, the ISGs are anchored by a *trans*-membrane domain and have a T_1/2_ of 3–6 hours [37]–[39]. Despite a copy number of ∼1×10^5^, the shorter ISG T_1/2_ suggests only a ten-fold difference between biosynthesis of VSG and ISG.

Here we address three questions. Firstly, as VSG is expressed at extreme levels, has the molecule evolved to fold efficiently? Secondly, are there discrete chaperone requirements between VSG and other surface molecules, specifically ISGs? Thirdly, how similar are the pathways present in trypanosomes to higher eukaryotes? We find a considerable chaperone requirement for both VSG folding and cell viability and that VSG is synthesized in considerable excess above levels required for the cell surface.

## Materials and Methods

### Ethics Statement

All cell lines for the present work were generated in house. Genetic modification to, and containment of, trypanosomes was authorized by the University of Cambridge Biological Safety and Ethics review panel.

### Bioinformatic Analysis and Open Reading Frame (ORF) Selection

*Trypanosoma brucei* 927 genome sequence data were searched at geneDB (http://www.genedb.org) [40]. The database was screened for ER chaperones and factors involved in ER quality control using; (i) an extensive list of query sequences corresponding to proteins identified in higher eukaryotes, comprising known chaperone and ER quality control families, (ii) a degenerate motif to identify KDEL/HDEL-related tetrapeptides at the predicted C-terminus of coding sequences, (iii) keywords to parse database annotations, and (iv) predictions of ORFs containing signal sequences and *trans-*membrane domains. BLAST searches were performed using the protein scoring matrix BLOSUM45. Initial returns were further filtered using: annotation information, reverse BLAST against the original query genome predicted ORF size being consistent with query, pfam prediction of conserved or expected domain architecture (where available), synteny with *Leishmania major* and *T. cruzi*, and phylogenetic reconstruction. This identified 28 ORFs for inclusion. Phylogenetic analysis was performed using Clustal X for initial alignment, manual editing in MacClade and then Mr Bayes (V3.2) run locally for 10^6^ generations using the mixed amino acid substitution model [41], PhyML at http://www.atgc-montpellier.fr/phyml/ with LG substitution model and 1000 bootstrap replicates [42] and RAxML at http://phylobench.vital-it.ch/raxml-bb/index.php with WAG substitution model and 1000 bootstrap replicates [43].

### Culturing of Bloodstream form *T. brucei*

*Trypanosoma brucei brucei* 427 Lister strain were cultured in HMI-9 complete medium (HMI-9 supplemented with 10% heat-inactivated FBS, 100 µg/ml penicillin, 100 µg/ml streptomycin and 2 mM L-glutamine) [22] at 37°C with 5% CO_2_ in a humid atmosphere in non-adherent culture flasks with vented caps. Cells were maintained at densities between 5×10^4^ and 2×10^6^ cells/ml. The single marker tetracyclin-inducible line was used for RNAi [44]. Plasmid constructs were maintained with G418 and/or hygromycin B, both at 2.5 µg/ml [22].

### Construction of RNA Interference Plasmids

Primers for amplification of RNAi targets were designed using RNAit [45] (http://trypanofan.path.cam.ac.uk/software/RNAit.html Table S1). Genomic DNA from Lister 427 cells was used as a template. RNAi fragments were PCR amplified using Taq DNA polymerase and cloned into the RNAi expression vector p2T7^TAblu^ and linearized with *Eam*1105I. All constructs were verified by standard sequencing methods.

### Transfection of Trypanosomes

3×10^7^ cells in log phase growth were harvested by centrifugation at 800 *g* for 10 minutes at room temperature and resuspended in 100µl AMAXA Human T Cell Solution at 4°C (Amaxa Inc.). 10µg of *Not*I-linearized p2T7 plasmid (in 5µl of water) was added to an AMAXA cuvette and immediately followed by 100µl cells. Transfection was performed in an AMAXA Nucleofector II and cells immediately transferred into a sterile flask prepared with 30ml pre-warmed HMI-9 (37°C) containing G418 at 2.5µg/ml. Hygromycin was applied to cultures six hours after transfection. Culture aliquots were distributed to three 24-well plates (undiluted, diluted 10-fold and 100-fold) and then incubated at 37°C, 5% CO_2_. Antibiotic-resistant transformants grew to saturation typically within 5–6 days. Cultures were expanded in flasks and continuously subcultured in the presence of antibiotics and analyses performed as soon as possible following. Cells were maintained in culture for a maximum of two months, and growth assays used to monitor continued RNAi sensitivity.

### Assessment of RNAi Impact on Proliferation

Following transfection and selection cells were counted using a Z2 Coulter Counter and adjusted to 5×10^4^ cells/ml in 20ml of HMI9. This 20ml culture was the divided into two 10ml cultures. One was induced with 1µg/ml tetracycline. The cultures were then counted at the same time each day and subcultured back to 5×10^4^ cells/ml (tetracycline was added fresh daily). Cultures where cell numbers fell below 5×10^4^ cells/ml were not subcultured on that day.

### RNA Extraction

1×10^8^ log phase cells were harvested at 3500 *g* for 10 minutes at 4°C and washed with ice-cold PBS and quick frozen in dry ice for one minute. In cases where severe proliferative defects were manifest, cells were harvested at the point where the proliferation defect was beginning to become significant. RNA was purified using the Qiagen RNeasy kit following the manufacturer's instructions. RNA quantity and purity was measured using a NanoDrop ND-1000 spectrophotometer and software (Thermo-Fisher).

### Quantitative Real-Time Polymerase Chain Reaction

2µg total RNA was diluted to 10µl with diethylpyrocarbonate (DEPC)-treated water and denatured at 70°C for 5 minutes. 15µl of a reaction cocktail was added (2.5µl dNTPs (25mM stock), 5µl 5× reverse transcription buffer (Invitrogen), 2µl of 100mM DTT, 0.5µl RNAseOUT (recombinant ribonuclease inhibitor, 40U/µl, Invitrogen), 2µl oligo dT (T_30_VN, 10µM stock), 0.5µl Superscript II Reverse Transcriptase (200U/µl Invitrogen), and 2.5µl DEPC-treated water) and incubated at 37°C for 1 hour, heat-inactivated at 90°C for 5 minutes and finally diluted to 200µl with DEPC-treated water. For qRT-PCR, 5µl of cDNA was used in a 25µl reaction including IQ-SYBR Green Supermix (BioRad) with 0.4µM gene-specific forward and reverse primers. qRT-PCR reactions were performed in white thin-wall polypropylene multiplate 96-well unskirted PCR plates (BioRad) sealed with microseal ‘B’ adhesive (BioRad). Reactions were performed in a BioRad MiniOpticon real time PCR detection system and included an initial denaturation at 95°C for 3 minutes, 40 cycles of 95°C for 30 seconds, 58°C for 30 seconds and then 72°C for 6 minutes (with a signal read at the end of each cycle), and a final melting curve to check fidelity from 60 to 95°C, with a signal read every 1°C. Gene-specific 20 base pair primers for each gene were designed using Primer3 (http://primer3.sourceforge.net) specified to amplify a ∼120 bp fragment (+/−10 bp) in the last kilobase of the 3′ end of the open reading frame and to avoid the region targeted by RNAi (Table S2). Primer pairs were validated *in silico* using Amplify V3.1.4 (http://engels.genetics.wisc.edu/amplify/) to minimize the probability of mispriming or formation of primer dimers and/or secondary structure.

### Western Blot Analysis

Cells were harvested at 800 *g* for 10 minutes and washed twice in ice-cold 1×PBS (Sigma). 1×10^7^ cells were heated in 100µl of 6× SDS boiling sample buffer [10% (w/v) glycerol, 100mM DTT, 3% (w/v) SDS, 0.01% (w/v) bromophenol blue and 50mM Tris–HCl (pH 6.8)] for 10 minutes at 95°C. 1×10^6^ cells per lane were loaded and resolved by SDS–PAGE on 12.5% SDS–polyacrylamide 10cm gels. Proteins were electrophoretically transferred onto nitrocellulose membranes (GE Healthcare) at 11V overnight in 190mM glycine, 25mM Tris-base, 20% (v/v) methanol using a wet transfer tank (Hoefer Instruments). Non-specific binding was blocked with Tris-buffered saline with Tween-20 (TBST) (137mM NaCl, 2.7mM KCl, 25mM Tris base pH 7.4, 0.2% Tween 20) supplemented with 5% freeze-dried milk for 2 hours at room temperature. Polyclonal rabbit anti-TbBiP serum (a kind gift of J. D. Bangs), polyclonal rabbit anti-VSG221 serum, polyclonal rabbit anti-ISG65 serum, polyclonal rabbit anti-ISG75 serum were used at 1∶10 000, 1∶5 000, 1∶5 000 and 1∶5 000, respectively. Incubations with commercial secondary anti-IgG rabbit horseradish peroxidase conjugates (Sigma) were performed at 10 000-fold dilution in TBST milk. Detection was by chemiluminescence with luminol (Sigma) on BioMaxMR film (Kodak).

### Densitometry

All fluorographs were scanned at 16-bit gray scale, and exposures selected to ensure that film was unsaturated. In most cases, the exposures in the figures represent overexposed versions of the same data used in quantification. Quantification and background subtraction were done with ImageJ (http://rsbweb.nih.gov/ij/).

### Indirect Immunofluorescence

3×10^7^ cells were harvested at 800 *g* at 4°C for 10 minutes and washed with ice-cold Voorheis's-modified phosphate-buffered saline (vPBS; PBS supplemented with 10mM glucose and 46mM sucrose, pH 7.6). The supernatant was aspirated, 250µl of 6% parmformaldehyde (in vPBS) and 200µl vPBS were added (∼3% final). Cells were fixed on ice for 10 minutes and applied onto poly-L-lysine coated glass slides (Sigma), previously sectioned with an ImmEdge Pen (Vector Laboratories, Inc.), for 30 minutes. For permeabilization, cells were incubated with 0.1% Triton-X-100 in PBS for 10 minutes at room temperature and washed three times for 5 minutes with PBS. Samples were blocked in 20% (v/v) FBS in PBS at 4°C overnight. Fixed cells were incubated with primary antibodies for 1 hour at ambient temperature, followed by three washes of 5 minutes each in PBS. Polyclonal rabbit anti-BiP serum and polyclonal rabbit anti-VSG221 serum were used at 1∶1000. Secondary antibodies were then applied for 1 hour at ambient temperature and washed as above (anti-mouse Oregon Green (Molecular Probes) at 1∶1000 and anti-rabbit Cy3 (Sigma) at 1∶1000). Samples were air dried, and coverslips were mounted using Vectashield mounting medium supplemented with 4′,6′-diamidino-2-phenylindole (DAPI) (Vector Laboratories, Inc.). Coverslips were sealed with nail varnish (Max Factor Inc.). Specimens were examined on a Nikon Eclipse E600 epifluorescence microscope fitted with optically matched filter blocks and a Hamamatsu ORCA charge-coupled device camera. Digital images were captured using Metamorph software (Universal Imaging Corp.) on a Dell computer running Windows XP (Microsoft Inc.), and the raw images processed using Adobe Photoshop CS4 (Adobe Systems Inc.) on a Macintosh computer (Apple).

### Proteosome Inhibitor Treatment

Lister 427 SMB cells were counted and adjusted to 3×10^5^ cells/ml in 450ml of HMI9. The 450ml culture was split into three 150ml cultures. One of the cultures was treated with 10µg/ml MG-132 (Calbiochem), the second culture with 20µg/ml MG-132, and the third a control. 1×10^7^ cells were collected from each culture after 0, 2, and 4 hour of incubation. Immediately after collection cells were harvested at 800 *g* for 10 minutes and washed in ice-cold PBS. Cell pellets were boiled in 100µl of a sample buffer and 10^6^ cell-equivalents per lane were used for Western blot analysis.

## Results

### Evidence for an Exocytic Quality Control System in Trypanosomes

VSG is a super-abundant antigen trafficked to the cell surface in an efficient manner, with kinetics suggesting a minimal period in the ER [27], [46]. We previously suggested the absence of a trypanosome unfolded protein response (UPR) on account of transcriptional inflexibility and the requirement for transcription factor splicing in activating the higher eukaryote pathway [22], [47]. However, either defective protein folding or N-glycosylation does lead to altered VSG trafficking and failure to reach the cell surface [29], [30] and the C-terminal GPI-signal peptide functions as an ER-retention signal [48], [49]. As ERQC is normally associated with proteasome-mediated turnover, we sought evidence of such a pathway in trypanosomes.

**Figure 2:**
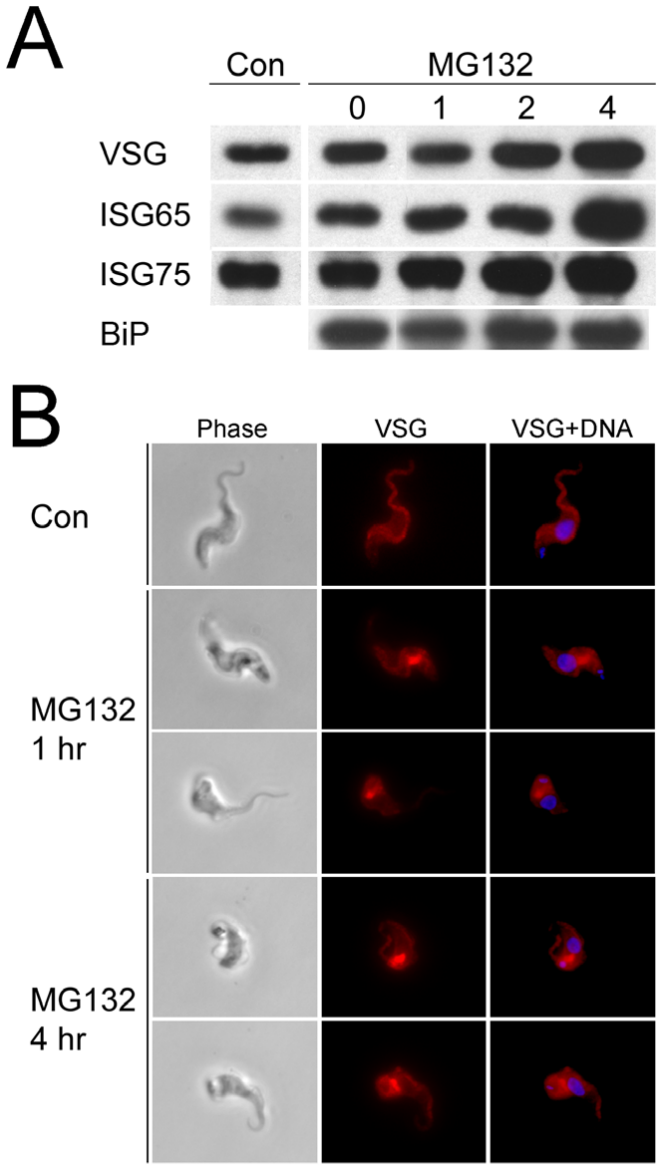
Proteasomal involvement in VSG turnover. Panel A: Western blot of cell lysates from trypanosomes exposed to 20µM MG-132 for the indicated times and probed for VSG221, ISG65 and ISG75, using polyclonal antibodies. “Con” indicates lysates from untreated cells, while MG132 lanes correspond to hours exposure to the compound. Lysates represent 1×10^6^ cell equivalents. Similar results were obtained with 10µM MG132, except that accumulation was less pronounced. The experiment was repeated three times with highly similar results. Equivalence of loading was monitored by reprobing of membranes with antibody to TbBiP. Panel B: Immunofluorescence analysis of trypanosomes either untreated (Con) or exposed to 20 µM MG123 for 1 and 4 hours. Following culturing cells were fixed, permeabilized and stained for VSG221 (red) and DNA (DAPI, blue). Note accumulation of VSG within the cell in the MG132-treated examples, which likely corresponds to aggresomes.

MG-132 is a proteasomal inhibitor effective against trypanosomes [50]. Unsurprisingly, cell viability was compromised following prolonged exposure and analysis was restricted to a maximum of four hours at which point where cells retained apparently normal morphology and motility (Figure 2 and data not shown). Western analysis detected increased levels of VSG after four hours in lysates from cells treated with 10µg/ml MG-132 (data not shown), while the effect was substantially more pronounced with 20µg/ml MG-132, where VSG levels were clearly increased after only two hours and with greater accumulation at four hours (Figure 2). We also analyzed the cell lysates for the invariant surface glycoproteins ISG65 and ISG75, and observed increased levels of both after four hours MG-132 exposure. Blotting with antibody to TbBiP revealed that expression of the ER marker was not significantly altered. These data indicate a significant accumulation of both GPI-anchored and *trans*-membrane domain proteins following proteasome inhibition. We addressed this further by analysis VSG location in MG-132 treated cells and found a substantial accumulation of VSG in perinuclear sites between the nucleus and kinetoplast, juxtaposed to the region of the cell containing the Golgi complex. These structures resembled an aggresome, an aggregate of polypeptides retrotranslocated from the ER that accumulate when proteasome activity is inhibited in mammalian cells [51].

Together this evidence suggests firstly an MG-132-sensitive turnover pathway for VSG and ISG, i.e. the proteasome, and secondly, that inhibition leads to accumulation of intracellular material, associated with the exocytic pathway and/or cytoplasmic aggresomes. Therefore both high abundance GPI-anchored and *trans*-membrane domain proteins are apparently synthesized to excess and at steady state the excess is degraded *via* the proteasome. These observations suggest a retrotranslocation QC mechanism, similar to higher eukaryotes, is present in trypanosomes [52].

### *In Silico* Identification of ERAD/ERQC Components in Trypanosomes

With experimental evidence for ERAD in *T. brucei* we sought to identify specific genes potentially involved in VSG biosynthesis and ERQC by searching the genome for orthologs and paralogs of genes that participate in protein folding in higher eukaryotes. We focused on chaperones of the HSP70/DNAj classes, proteins involved in glucosylation and recognition of N-glycans on nascent ER polypeptides and selected molecules of the protein disulphide isomerase (PDI) system (Table 1). We included Sec61, a component of the ER translocon, as a positive control, and two proteins, Lag1 and CERT, implicated in specific transport of lipids and/or GPI-anchored proteins. For reasons of practicality the target list excludes several PDI/thioredoxin-related and other factors previously analyzed in other contexts [53], [54].

**Table 1:** ER chaperones and ER quality control gene products in *Trypanosoma brucei*.

Accession (geneDB)	Annotation	Functional assignment	*T. brucei* status^1^
**HSP and DNAj**			
11.02.5450/5500^2^	BiP	Major ER chaperone	Orthologue
09.160.3090^3^	Hsp70	Chaperone	Paralogue (SS, degenerate HDEL)
09.211.1390	Hsp70	Chaperone	Paralogue
11.01.3110^4^	Hsp70	Chaperone	Paralogue
927.3.3580	Endoplasmin/GRP94/LPG3	Secretory pathway chaperone	Orthologue (SS, degenerate HDEL)
927.3.1430	DNAj	Chaperone	SS, DNAj domain
11.01.8480	DNAj	Chaperone	SS, DNAj domain
09.211.3680	DNAj	Chaperone	SS, DNAj domain
10.70.5440	DNAj	Chaperone	SS, DNAj domain
09.211.1550	Sec63	Part of DNAj family	Divergent orthologue [54]
**Glucosylation and lectins**			
	Calnexin	Quality control	Not found
927.4.5010/927.8.7410^2^	Calreticulin	Quality control	Orthologue
	ER glucosidase I	Quality control	Not found
10.05.0080	ER glucosidase II	Quality control	Orthologue
927.8.2910/2920/2930/2940^5^	EDEM	Quality control	Orthologue
927.3.4630	Glycoprotein glucosyltransferase I	Quality control	Orthologue
11.01.2470	Yos9	Quality control	Orthologue
11.02.1680	ERGIC53	Selective quality control	Orthologue
10.20.0130	Vip36	ERGIC53 paralogue	Orthologue
**Protein disulfide isomerase system**			
927.7.1300	ERp72-like	Cofactor with calnexin, PDI activity	Orthologue
10.6k15.2290	ERp57-like	Cofactor with calnexin, PDI activity	Orthologue
927.8.4890	Ero1	Oxidizes PDI	Orthologue
927.7.5790	ERp44-like	Complexes with Ero1, oxidoreductase	Orthologue
**GPI/lipid transport class**			
927.4.4740	Lag1/Dgt1	ER to Golgi transport of GPI proteins	Orthologue
	CERT	ER to Golgi transport of ceramides	Not found
**Translocon (control)**			
11.02.4100	Sec61	Part of ER translocation channel	Orthologue

Gene products were identified by searching the trypanosome genome database as described in methods.

1 Indicates presence of orthologue that fulfils criteria of reverse blast to higher eukaryote sequence, correct domain and sequence feature retention, or not found, i.e. fails criteria. Note that not found does not necessarily mean that a gene product with similar function to the higher eukaryote query sequence is not present. Paralogues indicates multiple distinct genes found, and based on sequence alone orthology cannot be unambiguously established.

2 BiP: Tb11.02.5450 and Tb11.02.5500 ORF sequences are identical. Calreticulin: Tb927.4.5010 (chromosome 4) and Tb927.8.7410 (chromosome 8) ORF sequences are identical.

3 Hsp70 (Tb11.01.3110) - no signal peptide; C-terminal ER retention motif is very degenerate (SSSL).

4 Hsp70 (Tb09.160.3090) - C-terminal ER retention motif is very degenerate (LKDLK LGE).

5 EDEM: cluster of four genes: Tb927.8.2910, 2920 and 2930 are near identical, while Tb927.8.2940 is C-terminally truncated, suggesting two EDEM paralogues; designated class I and II respectively. Two RNAi constructs were designed, EDEM A and B, which will target both isotypes due to high DNA sequence conservation over much of the ORF.

The DNAj family of chaperones are important factors in folding of nascent polypeptides. At least five mammalian ER lumenal forms are known (ERdj1-5) plus the mammalian ortholog of Sec63p, a component of the Sec61 translocon [55]. The trypanosome genome contains over 50 ORFs encoding potential DNAj-domain proteins, too many for the planned systematic analysis. Therefore, we parsed the DNAj ORFs for N-terminal signal sequences or signal anchors, feature of ERdj proteins from mammals and *S. cerevisiae*, reducing the number of candidates to fifteen. None contained a clear C-terminal [K/H]DEL-motif. We then performed BLAST with all ERdj family sequences from mammals and *S. cerevisiae*. *H. sapiens* ERdj1 did not return significant hits, while *H. sapiens* ERdj2, 3, 4 and 5 and *S. cerevisiae* Scj1 did recover sequences containing N-terminal signal sequences. We analyzed these DNAj candidates by phylogenetic reconstruction and reverse BLAST against *H. sapiens* and *S. cerevisiae*. Reverse BLAST confirmed assignment as DNAj-family ORFs, but due to the size and diversity of the DNAj families, orthologous relationships could not be unequivocally assigned as cytoplasmic, mitochondrial and ER members of the Hsp/DNAj family were returned. Phylogenetic reconstruction required removal of Tb09.211.1550 and ScJem1 due to extreme divergence. The final tree confirmed the weak relationships, with low to moderate support for a relationship for Tb09.211.3680 and Tb10.70.5440 to *H. sapiens* ERdj3 (Figure S1). Data from others supports assignment of Tb09.211.1550 as *T. brucei* Sec63 and was not investigated further [54]. These data suggest lineage-specific events within evolution of the trypanosome DNAj family, precluding unequivocal establishment of orthologs. However, based on these data and the confident prediction of ER targeting using PSORT II (http://psort.ims.u-tokyo.ac.jp/cgi-bin/runpsort.pl), we restricted analysis of DNAj proteins to Tb09.211.3680, Tb10.70.5440, Tb927.3.1430 and Tb11.01.8480 (Table 1).

Five gene products are annotated as HSP70, or HSP70-related and also possessing a signal sequence in the trypanosome genome. Two ORFs encode TbBiP (Tb11.02.5450/5500) and the remaining are experimentally uncharacterized; Tb09.160.3090, Tb09.211.1390 and Tb11.01.3110 (Figure S2). In later genome assemblies/datasets, Tb11.01.3110 became annotated as lacking a signal sequence, confirmed with PSORT II. This region of the genome appears unstable as evidenced by loss of syntenic relationships between trypanosomes, abnormally sized intergenic regions and the presence of a short gene fragment (Tb11.01.3100) in assembly version 4 bearing no relationship to Hsp70, in contrast to earlier annotation.

Searches for the ERQC/glucosylation system were straightforward as these gene products are not, in the main, part of extensive paralogous families. We could confidently identify orthologs for most queries except calnexin and ER glucosidase I. The latter confirmed previous studies [56], and detection of glucosyltransferase I also confirmed an earlier analysis [33]. Interestingly, despite the absence of a Glc_3_Man_9_ precursor glycan from trypanosomatid nascent polypeptides [32], an ERQC system based on monitoring of protein-folding *via* transient reglucosylation cycles can be reconstructed *in silico*. Further, this system includes the PDI-related factors ERp52 and ERp72, which interact with calnexin/calreticulin; this system, together with the Ero1/ERp44 complex is fully represented (Table 1). Additionally, a cluster of ER degradation-associated mannosidase-related (EDEM) genes were found; Tb927.8.2910, Tb927.8.2920 and Tb927.8.2930 are extremely similar to each other, while one ORF, Tb927.8.2940, is truncated at the C-terminus, suggesting multiple EDEM proteins (Figure S4). We designated these EDEM orthologues as class I and II respectively, but due to high similarity it was not possible to target them individually. Further, the EDEM cluster contains a potential pseudogene and is possibly misassembled. Two RNAi constructs were used to target this ORF cluster, EDEM A and B. Finally, we identified an orthologue for Lag1 (927.4.470), but not for CERT.

Few of the selected gene cohort exhibit differential expression between bloodstream and procyclic forms [22]. Calreticulin (Tb 927.4.5010) is moderately upregulated in cultured bloodstream forms compared to trypanosomes grown in rodents, while ERp57 (Tb10.6k15.2290) is downregulated by tunicamycin treatment. Tb927.7.5790 (ERp44) is increased in the bloodstream form when compared with insect stages. However, none of these changes is easily explained as a response to altered environment or stress in the absence of a coordinated transcriptional shift and there is little evidence for major transcriptional remodeling of ER chaperones during development. Overall, *in silico* analysis indicates a complex, conserved representation of ER folding and QC gene products and that dominance of the exocytic pathway by VSG and VSG-related proteins, i.e. ISGs, has not resulted in major secondary losses. The absence of calnexin is the major secondary loss of note, but as the knockout in *S. cerevisiae* is viable [57] and as there is considerable redundancy between calnexin and calreticulin, the functional significance is unclear.

### Screening Trypanosome ER Protein Folding

**Figure 3:**
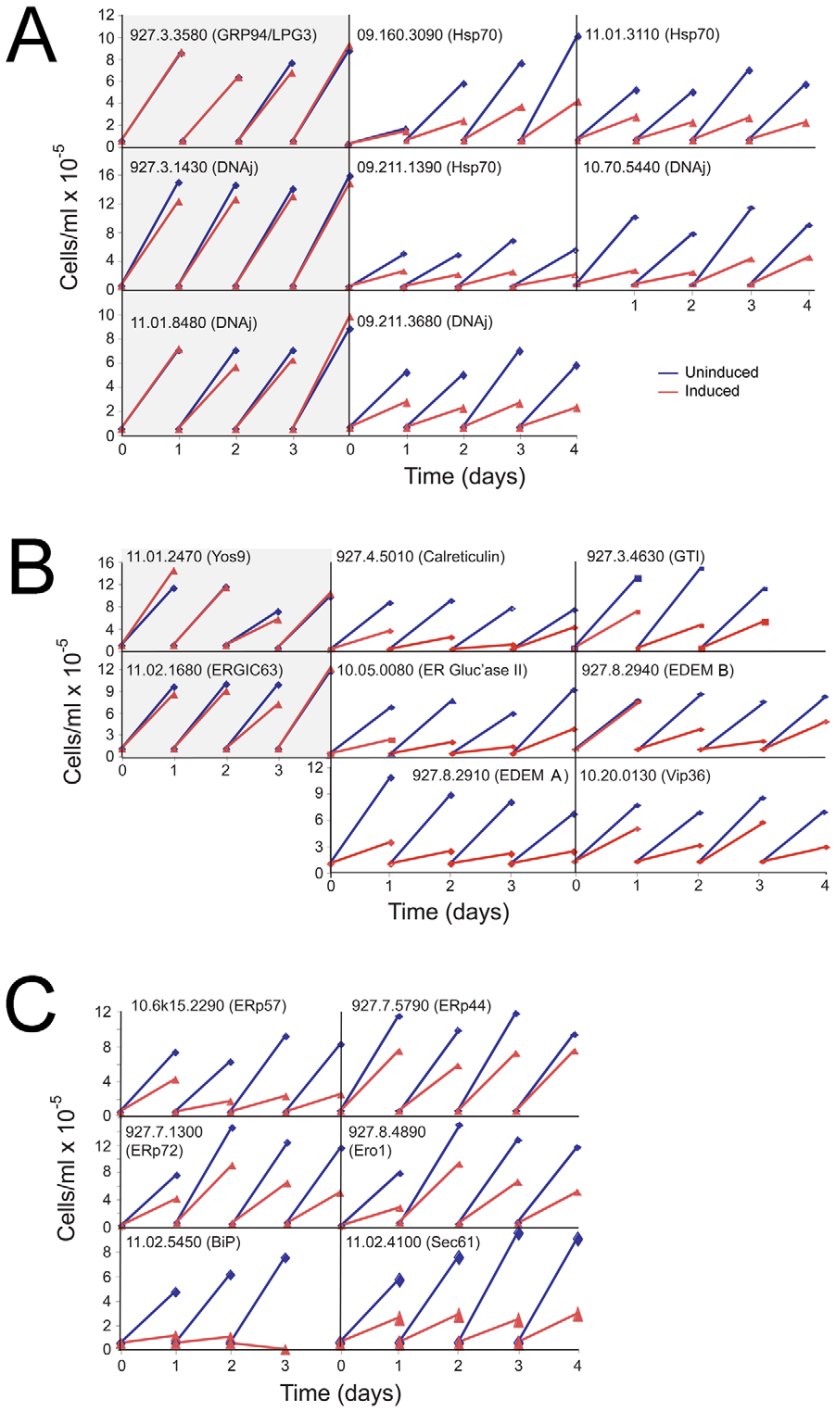
Influence of RNAi on trypanosome replication. Cells transformed with the p2T7 RNAi plasmid harboring an insert corresponding to the indicated gene, were analyzed for the effects of RNAi on replication. Cell numbers were determined daily, and cultures diluted as necessary (see methods). Panel A; HSP class, Panel B; Lectin/glycosidase class, and Panel C; others. Blue lines are data from uninduced cultures and red lines from induced. A single data set is shown, which is representative of three or more independent analyses on separate clones. Data for Tb927.4.4740 (Lag1) are not shown. Tb11.02.4100 (Sec61) and Tb11.02.5450 (BiP) are included as positive controls. RNAi clones scored as having normal growth are on a gray background. Note that in some instances the uninduced cultures appear to grow at slower rate when compared to untransfected (not shown) or cells harboring an RNAi construct with no apparent replication impact.

We selected a total of 28 ORFs for analysis (Table 1), encompassed by 24 distinct RNAi constructs due to identical sequence present in BiP, calreticulin and EDEM paralogs. We initially screened for proliferative defects as prevention of VSG biosynthesis results in growth arrest [31], and folding pathways are likely central to cell viability (Figure 3). All experiments were conducted using the Lister 427 328.114 (single marker bloodstream form (SMB)) cells [44] and proliferation monitored over four days. We applied criteria that a proliferation defect must manifest at least a 20% decreased cell number on two consecutive days. Surprisingly 68% of the RNAi lines exhibited met the criteria (Tables 2 and 3), higher that the 35% found by an earlier systematic screen [58]. Several RNAi lines displaying strong proliferative defects also replicated less well in the uninduced state (Figure 3), probably from incomplete repression of the RNAi construct. The high proportion of ORFs required to support normal morphology and replication was seen across the three categories of chaperone (Table 2), and suggests a surprising absence of redundancy within the cohort. Several DNAj and HSP proteins are clearly nonredundant as individual knockdowns compromised morphology, VSG biosynthesis and proliferation, but was specific as only the ERdj genes most similarity to *Homo sapiens* ERdj3 generated the VSG phenotype (Figure S1). The remaining DNAj ORFs in that cohort are divergent and probably either redundant or play distinct roles.

**Table 2:** Summary of phenotypic effects obtained by RNAi of *Trypanosoma brucei* ER chaperone candidates.

Function	Number^1^	Proliferation^2^	Morphology^2^	ER^2^	Increase in VSG abundance^2^
Chaperone/HSP	8	63	63	63	38
Calreticulin/redox	11	80	80	80	72
Post-ER lectins	2	50	50	50	50
GPI/sphingolipid	1	0	0	0	0
**Total**	**22**	**68**	**68**	**68**	**55**

Each ORF was subjected to suppression using RNA interference, and the effect on proliferation, cell morphology, ER morphology (ER) and the presence of increased VSG abundance monitored as described in methods and the text. ORFs are grouped in functional classes, using the same categories as in Table 1.

1 Total number of distinct ORFs analysed; number collapsed to one for multiple copies targeted by same construct.

2 Data for phenotype are expressed as percent of total number of ORFs tested.

**Table 3:** Phenotypic classification of defects obtained following suppression of expression under RNAi.

Accession (geneDB)	Annotation	Proliferation^1^	Morphology^2^	Vesiculated ER	Swollen ER	Multinucleated	Increase in level of		
							VSG	ISG65	ISG75
11.02.5450/5500	BiP	+	+		++	++	−		
09.160.3090	Hsp70 (Imported to ER)	+	+	+	−	++	+	+	−
09.211.1390	Hsp70 (Imported to ER)	+	+	−	−	−	−	−	−
11.01.3110	Hsp70 (cytosolic)	+	+	++	++	−	−	−	−
927.3.3580	GRP94/LPG3	−							
927.3.1430	DNAj	−							
11.01.8480	DNAj	−							
09.211.3680	DNAj	+	+	−	+	+	++	++	+/−
10.70.5440	DNAj	+	+	−	+	−	++	+/−	+/−
927.4.5010/927.8.7410	Calreticulin	+	+	+/−	++	++	+	+/−	+/−
10.05.0080	ER glucosidase II	+	+	−	+/−	++	++	−	−
927.8.2910/2920/2930/2940	EDEM A	+	+	−	+	++	+		
	EDEM B	+	+	−	+/−	++	+		
927.3.4630	Glycoprotein glucosyltransferase	+	+	−	+/−	+/−	++	−	−
11.01.2470	Yos9	−							
11.02.1680	ERGIC53	−							
10.20.0130	Vip36	+	+	−	+	+	++		
927.7.1300^3^	ERp72	+	−	−	−	−			
10.6k15.2290	ERp57	+	+	−	+	++	++	+/−	+/−
927.8.4890	Ero1	+	+	−	+/−	+	−		
927.7.5790	ERp44	+	+	−	+	++	+		
927.4.4740	Lag1/Dgt1	−							
11.02.4100	Sec61	+	+	−	++	++	+	+	+

All experiments were conducted in bloodstream stages and analysis of cells performed after one and two days induction.

1 Proliferation score: +; greater than 20% proliferation defect observed on at least two consecutive days.

2 Morphological and protein expression changes are scored as follows: ++; strong (expression level at least twice control, abnormal morphology in more than 50% of cells and severe), +; moderate (expression level up to twice control, morphology in up to 50% of cells and obvious), +/−; mild (expression level changes less than twice control, morphology in fewer than 50% of cells and not pronounced), and −; no detectable change. Blank; no data.

3 Tb927.7.1300 (ERp72) – this cell line has not been analysed by IFA or Western as positive clones could not be expanded.

**Figure 4:**
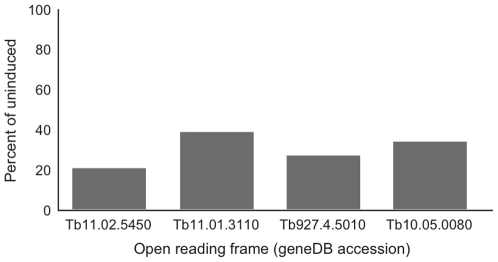
Validation of selected RNAi knockdowns using qRT-PCR. RNA was extracted from cultures at 24 hours following induction, and the level of mRNA corresponding to the knockdown target compared to uninduced controls. Data were normalized against tubulin mRNA. See methods for experimental details. ORFs are designated by their geneDB accessions.

**Figure 5:**
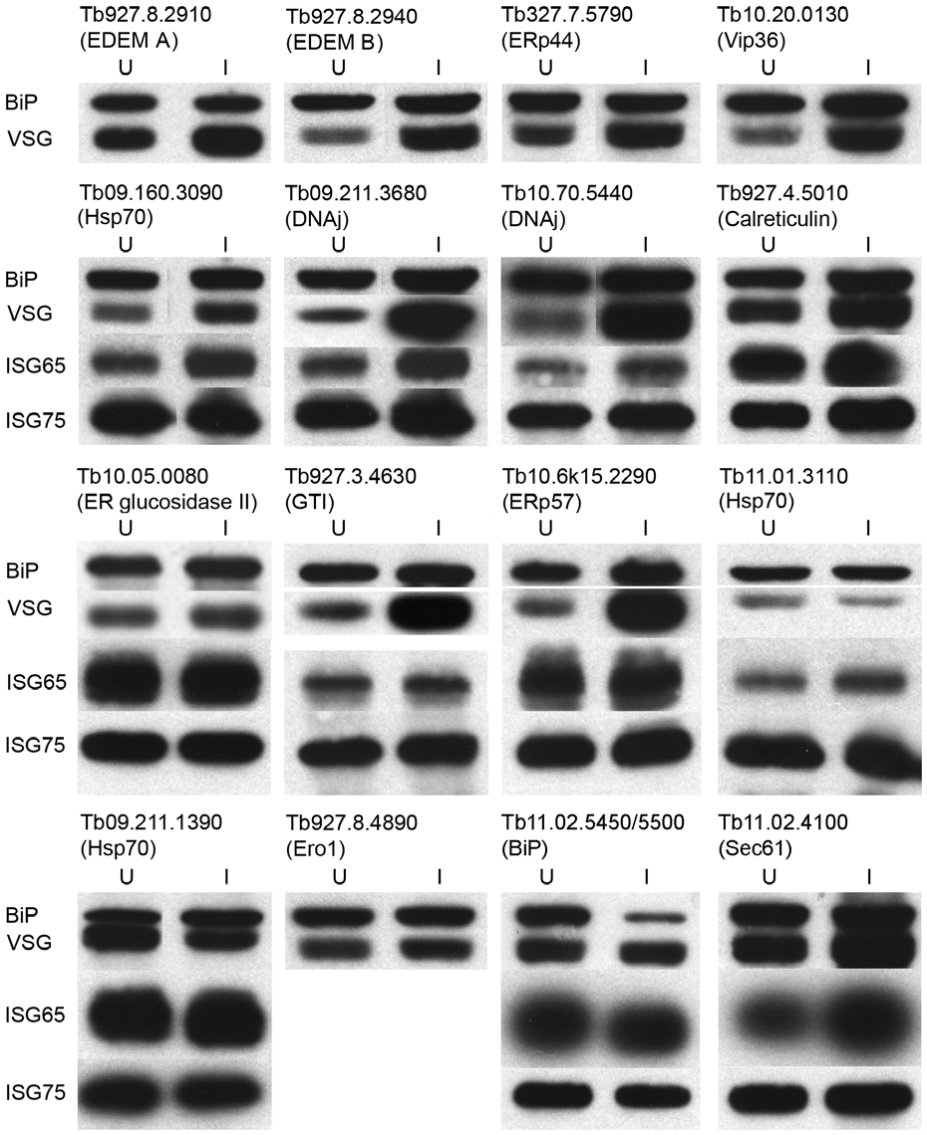
Expression of major antigens in trypanosomes following RNAi. Western blot analysis of whole cell lysates of cells induced for RNAi against various ORFs. Typically RNAi was performed for 24–48 hours and cells lysed in Laemmli sample buffer prior to fractionation and transfer to membrane. Antigens were detected as described in methods and quantitated by densitometry. Data are representative of at least two separate inductions. Lane images have been manipulated for presentation purposes; all lanes for each RNAi were derived from the same membrane.

We validated ∼15% of the RNAi knockdowns by quantitative RT-PCR using β-tubulin as internal control (Figure 4). All ORFs demonstrated significant loss of mRNA, between ∼60%–80% decrease, indicating specificity and efficiency. As the validated RNAi lines also exhibited proliferative defects, it remains possible that some of the ORFs with normal proliferation are the result of failure to sufficiently impact mRNA levels. However, given the high frequency of proliferative defects we chose not to investigate these further, but rather continue analysis of the positive cohort. Additionally, both constructs targeting the EDEM cluster generated very similar phenotypes, providing additional validation (Table 3, Figures 3 and 5).

### Effects of Suppression on VSG and ISG Expression

**Figure 6:**
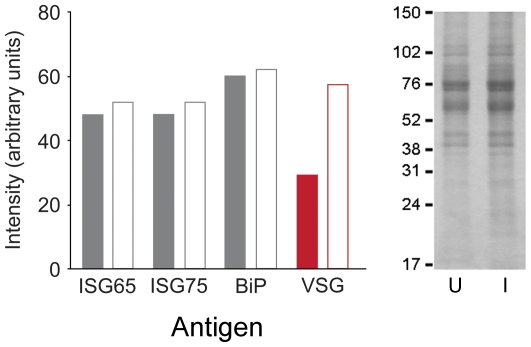
Validation of Western blot analysis. Cell lysates from induced and uninduced cultures harboring the Tb10.70.5440 (DNAj) RNAi construct were fractionated by SDS-PAGE. Duplicate samples were separated for Coomassie and Western analysis. Left panel: Western blot quantitation using ECL and densitometry from X-ray film for various antigens in cells uninduced and induced for Tb10.70.5440 RNAi (closed and open bars respectively). Data are normalised to total protein as determined from the right hand panel. Note data are from a separate experiment to Figure 5, and are highly comparable. Right panel; Coomassie stained SDS-PAGE analysis of trypanosome lysates. Numbers indicate molecular weights of co-electrophoresed standards, and U and I indicate uninduced and induced for Tb10.70.5440 RNAi respectively. The entire region of the gel between 17 and 150 kDa was scanned for densitometric normalisation. A separate experiment conducted on Tb09.11.3680, a second DNAj-related ORF, demonstrated highly similar results, except that ISG65 was also significantly increased (Figure 5 and data not shown).

To directly address effects of RNAi on protein fate, we performed Western blotting of whole cell lysates prepared from cells induced for RNAi. We probed for BiP (loading control), VSG, ISG65 and ISG75 and observed increased VSG immunoreactivity in twelve of sixteen lysates, with the effect distributed across several classes of gene product (Figure 5). These data suggest that VSG accumulates in these cells. The increase was validated by normalization against total protein as determined by Coomassie blue staining of Tb10.70.5440 RNAi cell lysates (Figure 6). Significantly we found that levels of TbBiP protein were decreased in the BiP RNAi line (Tb11.02.5450/5550), confirming knockdown. However, there was no corresponding increase to detectable VSG in these cells, probably due to the rapid onset of lethality.

A pronounced effect was observed for only one (Tb09.160.3090) of three ER-targeted Hsp70 genes, indicating differential effects on VSG. RNAi against a cytosolic Hsp70, Tb11.01.3110, as expected, did not alter VSG levels, despite significant and wide-ranging phenotypic affects (Table 3). In a subset we observed increased ISG65, specifically Hsp70 (Tb09.160.3090) and two DNAj proteins (Tb09.211.3680 and Tb10.70.5440). There was a modest increase in ISG65 in three further RNAi lines, calreticulin (Tb927.4.5010), ERp57 (Tb10.6k15.2290), and Sec61 (Tb11.02.4100). Finally, the effects on ISG75 were very minor. These data indicate that not all factors affecting VSG levels also manifest as accumulation of ISG polypeptides, suggesting either differential chaperone/QC requirements or failure to detect ISG defects due to differential turnover rates [39]. Further, while there is correlation between defective proliferation and VSG accumulation, *albeit* with some exceptions (e.g. BiP), this does not hold for ISGs. ISG65 expression is nonessential for *in vitro* culture (MC, unpublished data), whereas VSG expression is required for continued viability. Taken together these observations suggest that VSG biosynthesis has a greater requirement for ER factors, which could be anticipated for the major biosynthetic trypanosome ER protein product. Overall these data identify increased VSG protein level as a common phenotype following suppression of factors involved in ER-based folding or QC. Importantly this indicates that VSG is made in significant excess, fully consistent with the observations from proteasome inhibition.

### Effect of Suppression on Cell Morphology

**Figure 7:**
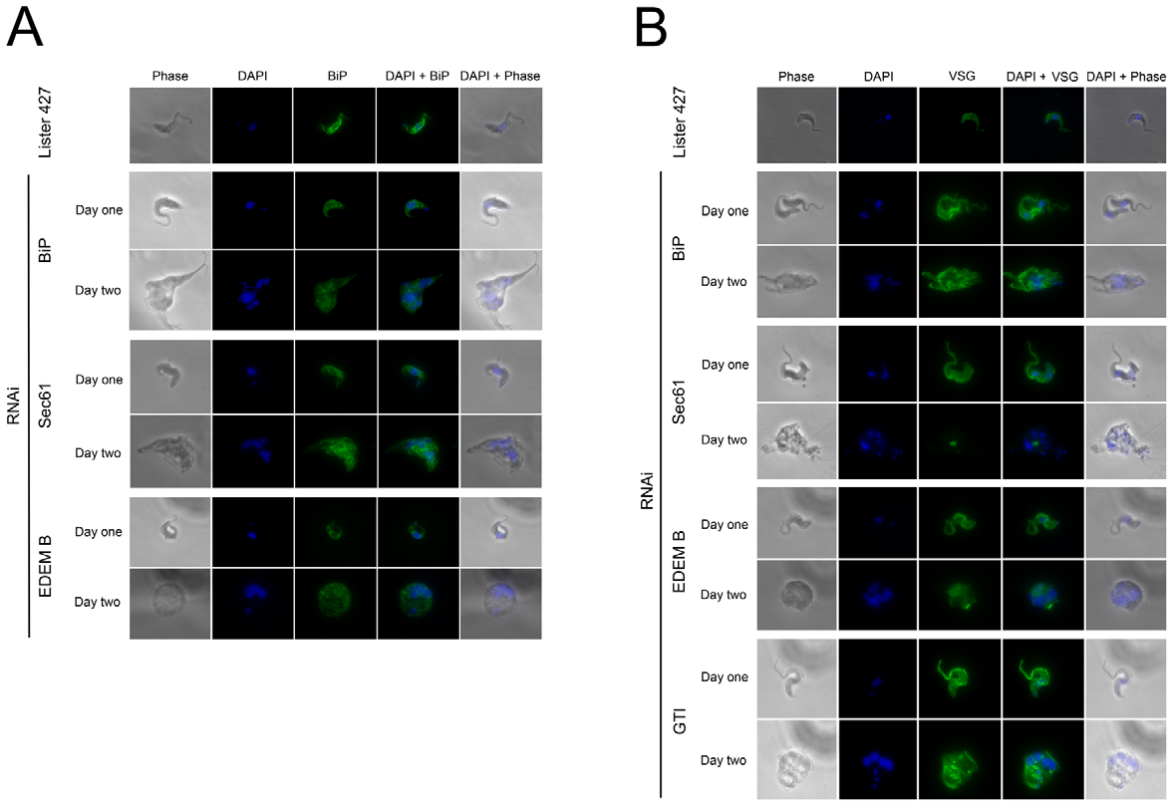
Morphological consequences of RNAi for ER folding factors. Panel A: Cells induced for RNAi stained for BiP (green) and DNA (DAPI, blue). Corresponding phase contrast and merged images are also shown. SMB is the parental line, stained for BiP as negative control. Panel B: As for panel A, but costaining for VSG221 (green). RNAi targets are designated by abbreviated gene name. Selected images are shown; the full dataset is provided as zip archive.

To monitor intracellular accumulation of VSG and ER morphology abnormalities, cells were analyzed on day one and two post induction and stained for VSG and BiP (Figures 7 and S3). For all Hsp and DNAj factors we observed morphological abnormalities. Cells were malformed and in some cases puncta containing BiP together with swollen ER were found. Of the HSP genes analyzed, Tb09.211.1390 and Tb09.160.3090 were predicted to be localized to the ER lumen (Figure S2). Knockdown led to severe proliferative and morphological defects, but interestingly only Tb09.160.3090 led to distortion of the ER and accumulation of VSG and ISG. Further, the third HSP70 RNAi analyzed, Tb11.01.3110, predicted as cytoplasmic and included as a negative control, also produced proliferative and morphological defects. Significantly while no effect on VSG levels were observed there was a very pronounced ER morphological defect, suggesting potential roles for cytoplasmic chaperones on the trypanosome ER. For the DNAj cohort, we examined four gene products, two of which are related to ERdj3, a defined ER chaperone (Figure S1). RNAi against both of these genes, Tb09.211.3680 and Tb10.70.5440, generated clear VSG and ISG accumulation, suggesting non-redundant functions. The remaining genes, Tb927.3.1430 and Tb11.01.8480, produced no detectable phenotype; this may be due to redundancy, the absence of a significant function in the bloodstream form, a nonessential role or failure to suppress the gene products sufficiently. These data suggest gross defects leading to mislocalization of the major chaperone together with additional effects manifest throughout the cell. There was also evidence for accumulation of VSG in several lines, consistent with failure to complete folding/QC processes. The presence of multinucleated cells in some knockdowns is also consistent with the requirement for ongoing biosynthesis and exocytosis of membrane components, including VSG, for completion of cytokinesis and the cell cycle. However, mitosis appeared to be comparatively unaffected as DNA replication and nuclear segregation were essentially normal.

The calreticulin/glucosylation system demonstrated particular sensitivity to RNAi, and all genes, including the associated factors ERp57 and ERp44 produced detectable VSG accumulation. We also observed multinucleated cells, BiP and VSG aggregates or puncta in calreticulin and glucosyltransferase system knockdowns. The overall defects were rather similar to the Hsp/DNAj system, suggesting that this is a phenotype associated with disruption of ER protein folding. Again, defects in cytokinesis were found, without an obvious block to S-phase or mitosis. For PDI-associated gene products we also found a similar phenotype, with the exception of no obvious morphological defect for ERp72 (Tb927.7.1300). Both ERGIC-53 and VIP36 are oligomannose-recognizing L-type lectins, and knockdown suggests a role for VIP36 in VSG biosynthesis, but not ERGIC-53 [59], [60]. The involvement of ERGIC-53 in protein folding is limited in higher eukaryotes and despite a role in maintaining the structure of the Golgi complex, most anteriorgrade transport is ERGIC-53 independent [61]. Hence absence of an obvious requirement by VSG is not unprecedented.

Finally, the Sec61 RNAi phenotype was very severe, with extremely disrupted cells consistent with Sec61 as a major component of the ER translocon. Knockdown likely disrupts co- and post-translational translocation plus retrotranslocation of ERAD substrates. Remarkably some increase in VSG level was detected even here (Figure 5). Disruption of ER protein folding appears to result in a similar phenotype for many factors, including swollen ER, BiP and/or VSG-positive puncta, but with little evidence of mitotic defects. While these data indicate a common morphological end point, and do not allow discrimination between the different ER pathways, they validate the growth data and further confirm the need for multiple gene products for VSG folding.

## Discussion

The trypanosome surface is unusual as ∼90% of cell surface protein is GPI-anchored VSG. We wished to determine if the bloodstream form trypanosome exocytic pathway has become specialized for specific production of VSG and to consider if VSG is in some manner adapted for efficient folding, to reduce burden on the trypanosome ER. Given that there is currently little known concerning ERQC in trypanosomes, initially we sought evidence for the presence of proteasome-dependent ER-associated degradation. Inhibition of the proteasome increased VSG levels and resulted in production of intracellular puncta or aggresomes, indicating the likely presence of a conventional ERAD pathway [3], [6]. As VSG is GPI-anchored we mainly analyzed gene products associated with the ERAD-L pathway, but included several general factors, e.g. Yos9, important for recognition of malfolded proteins, and proteins involved in selective post-ER transport, specifically VIP36, ERGIC-53 and Las1. Finally, we used TbBiP and TbSec61 as positive controls. TbBiP and TbSec61 knockdown led to rapid and major cellular defects, consistent with central roles in folding and translocation pathways. While more detailed dissection of the roles of individual gene products is clearly a necessity for precise and mechanistic conclusions, several significant findings emerge from this analysis.

Firstly, there is a considerable burden on the trypanosome ER for folding and export of VSG. Hence, a paradigm whereby VSG evolved to fold efficiently appears unlikely, based on the requirement for both a large cohort of chaperones and evidence of quality control monitoring. The presence of an ERQC system is strongly supported by detection of excess VSG synthesis. VSG accumulation was observed both in multiple knockdowns and following inhibition of the proteasome. As VSG probably evolved comparatively recently from non-variable surface antigens, structural constraints from that earlier role may remain. We estimate that VSG is synthesized in 2–3 fold excess by Western analysis. As VSG represents ∼10% of cell protein, this excess is of the order of 20–30% of protein synthetic output of the cell, assuming no other polypeptides are made to considerable excess. This requirement places a significant, and previously unappreciated, energetic cost to the parasite. Therefore, VSG is not streamlined for rapid, chaperone-independent folding. By comparison, the CFTR channel is considered very inefficient, with 10% attaining the cell surface, but the data here suggests remarkably that VSG is only three-fold better.

Secondly, a consensual phenotype emerged when ER function was perturbed. Morphological defects encompassing both the ER and cell body, defective cytokinesis and nuclear segregation and internal accumulation of VSG were frequent. This suggests that chaperone suppression lead to a slower rate of exit of VSG from the ER and accumulation of folding intermediates or terminally misfolded VSG. Further, onset of proliferative defects was rapid and likely occurring within one or two cell cycles as in many lines not even a doubling of cell numbers after 24 hours was achieved. Therefore there is a critical requirement for ongoing synthesis of many ER chaperones. Significantly the phenotype we obtained with the chaperone knockdowns is highly similar to that for GPI8, the *trans*-amidase responsible for addition of the GPI-anchor to VSG and other polypeptides [62], which suggests that this is the generic defect obtained when ER function in *T. brucei* is significantly compromised.

Thirdly, ISG accumulation was not found in all cases where VSG accumulated, and usually ISG was less profoundly affected than VSG. This may be due to the lower biosynthetic levels of ISGs and/or differential chaperone requirements. Significantly, ISG accumulation was never observed in the absence of VSG accumulation. However, the initial screen used proliferation as an assay, and ISG65 expression is not required for normal proliferation in culture (MC, unpublished data). More detailed study is clearly needed to document specific chaperone requirements.

Fourthly, the phenotype arising from suppressing ER factors is distinct from direct VSG knockdown [31]. RNAi directed against VSG results in discrete cell cycle arrest post-mitosis and around the time when cytokinesis initiates. To our knowledge this remains the sole example of such a discrete cell division phenotype in *T. brucei*. Why are these two RNAi phenotypes so distinct? A possible explanation is that the RNAi knockdowns described here affect more than just VSG itself and hence could result in failure to produce additional factors required for cell cycle arrest in the absence of sufficient VSG.

Fifthly, we never observed significant changes to the levels of TbBiP, except when TbBiP itself was targeted. This is further evidence against a conventional unfolded protein response in trypanosomes [22]. Despite the absence of an UPR, both *in silico* and functional data indicate a complex ERQC system in trypanosomes, requiring participation of many higher eukaryote orthologs. This analysis extends an earlier comparative genomics study which identified ERQC genes in a range of taxa, suggested an ancient origin for these systems and hinted that trypanosomes likely possess an ERQC pathway [56]. Significant divergence in these pathways is apparent between taxa however. For example, in *Arabidpsis thaliana* there is both an ERQC system and conservation of transcriptional pathways mediating the UPR [63]–[66]. By contrast multiple ERAD components are apparently targeted to a non-ER compartment, the apicoplast, in *Plasmodium falciparum* [67]. Divergence in the early N-glycosylation pathway in trypanosomes also indicates evolution of unusual or unique ER-based mechanisms within this lineage [68].

Sixthly and finally, the appearance of a phenotype for such a large cohort of chaperones indicates both a requirement for these factors and, from evidence for increased VSG levels in many cases, a direct role in VSG ERQC. Therefore, we conclude that, despite dominance of the exocytic pathway by VSG and VSG-related proteins, there is little evidence for simplification in the trypanosome chaperone requirement, and *T. brucei* retains a sophisticated, flexible and complex folding environment within the ER. The trypanosome exocytic pathway has not become specialized for specific production of VSG and nor is VSG adapted for efficient folding

## Supporting Information

Figure S1 Phylogenetic reconstruction of part of the DNAj family. Sequences for representative members of the DNAj family were retrieved from the NCBI nr database to represent the major Opistokhonta (animals and fungi) ER chaperone families plus Sec63 (*H. sapiens*, black, *S. cerevisiae*, blue). Searches of the *T. brucei* genome database using BLAST using the higher eukaryote sequences returned as most significant the four sequences shown here in by geneDB accession number (red) as well as Tb09.211.1550 (data not shown). All other sequences were rejected based on sigificantly lower expect values, excessive or very small predicted polypeptide size or reverse BLAST failure (frequently demonstrating orthology to mitochondrial DNAj proteins). Sequences were aligned in Clustal, manually edited in MacClade and subjected to phylogenetic analysis. Initial rounds demonstrated that Tb09.211.1550 was highly divergent and was removed. Further rounds of reconstruction resulted in the tree shown. Values at the internodes are bootstrap/bootstrap/posterior probability for RaXML, PhyML and Mr Bayes reconstructions. Data suggest that Tb09.211.3680 and Tb10.70.5440 are orthologs of ERdj3. The remaining two sequences are either trypanosome-specific or orthologs to DNAj proteins not included in the present analysis.
(2.54 MB TIF)

Figure S2 Phylogenetic reconstruction of part of the trypanosome Hsp70 family. Sequences for representative members of the trypanosome Hsp70 family were retrieved from geneDB. Sequences were aligned in Clustal, manually edited in MacClade and subjected to phylogenetic analysis. Gene products in red were analysed. Values at the internodes are bootstrap/bootstrap/posterior probability for RaXML, PhyML and Mr Bayes reconstructions. Annotations based on BLAST similarity to sequences at NCBI nr database and PSORT II are also provided. Note that most of these annotations should be considered tentative.
(3.19 MB TIF)

Figure S3 Immunoflurescence microscopy data archive. Data are shown for cells at one or two days post induction for RNAi for the indicated open reading frame. Cells were fixed, stained for either BiP or VSG (green) and counterstained for DNA using DAPI. Example images are binned according to frequency of the morphology observed. Frequent; >70%, common; 10–25%, rare; <5%. In all instances several hundred cells were analysed per gene product and representative images are shown for each category and time. Inductions were performed at least twice for each gene product with similar results. Data are available to download from http://homepage.mac.com/mfield/lab/PDFs/Field%20et%20al%202010%20supp%20data.pdf.
(268.15 MB ZIP)

Figure S4 Clustal aligmnents for predicted amino acid and DNA sequences of EDEM ORFs from *T. brucei*. Sequences corresponding to geneDB accessions Tb927.8.2910, Tb927.8.2920, Tb927.8.2930 and Tb927.8.2940 were aligned with Clustal X using default parameters.
(0.06 MB DOC)

Table S1 Primers for p2T7 RNAi constructs. Primer sequences are given 5′ to 3′, and the corresponding gene is designated by the geneDB accession number, except for VSG MITat1.2 where the NCBI accession is given.
(6.53 MB TIF)

Table S2 Primers for qRT-PCR transcription analysis. Primer sequences are given 5′ to 3′, and the corresponding gene is designated by the geneDB accession number.
(6.53 MB TIF)
