# Supplementary figures and images for: Chaperone Requirements for Biosynthesis of the Trypanosome Variant Surface Glycoprotein
Source: PLoS One. 2010 Jan 5;5(1):e8468. doi: 10.1371/journal.pone.0008468 (PMC2797082; doi:10.1371/journal.pone.0008468)

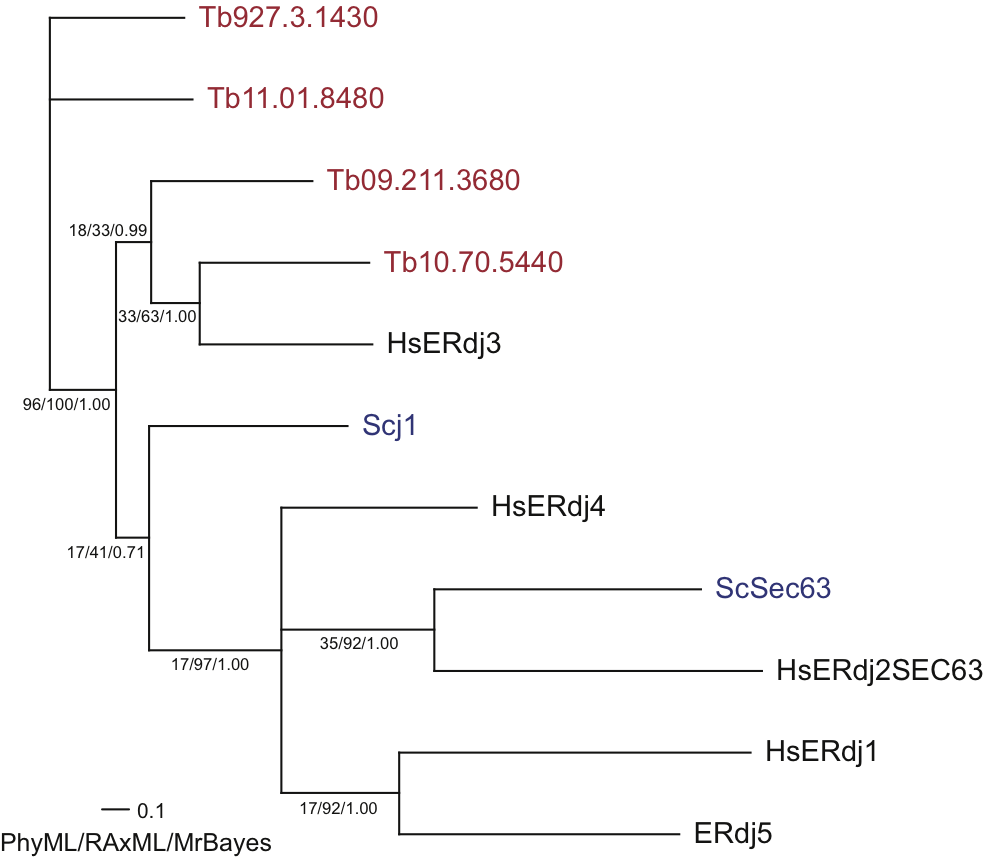

Supplement: Figure S1 — Phylogenetic reconstruction of part of the DNAj family. Sequences for representative members of the DNAj family were retrieved from the NCBI nr database to represent the major Opistokhonta (animals and fungi) ER chaperone families plus Sec63 (H. sapiens, black, S. cerevisiae, blue). Searches of the T. brucei genome database using BLAST using the higher eukaryote sequences returned as most significant the four sequences shown here in by geneDB accession number (red) as well as Tb09.211.1550 (data not shown). All other sequences were rejected based on sigificantly lower expect values, excessive or very small predicted polypeptide size or reverse BLAST failure (frequently demonstrating orthology to mitochondrial DNAj proteins). Sequences were aligned in Clustal, manually edited in MacClade and subjected to phylogenetic analysis. Initial rounds demonstrated that Tb09.211.1550 was highly divergent and was removed. Further rounds of reconstruction resulted in the tree shown. Values at the internodes are bootstrap/bootstrap/posterior probability for RaXML, PhyML and Mr Bayes reconstructions. Data suggest that Tb09.211.3680 and Tb10.70.5440 are orthologs of ERdj3. The remaining two sequences are either trypanosome-specific or orthologs to DNAj proteins not included in the present analysis. (2.54 MB TIF) [file pone.0008468.s001.tif]

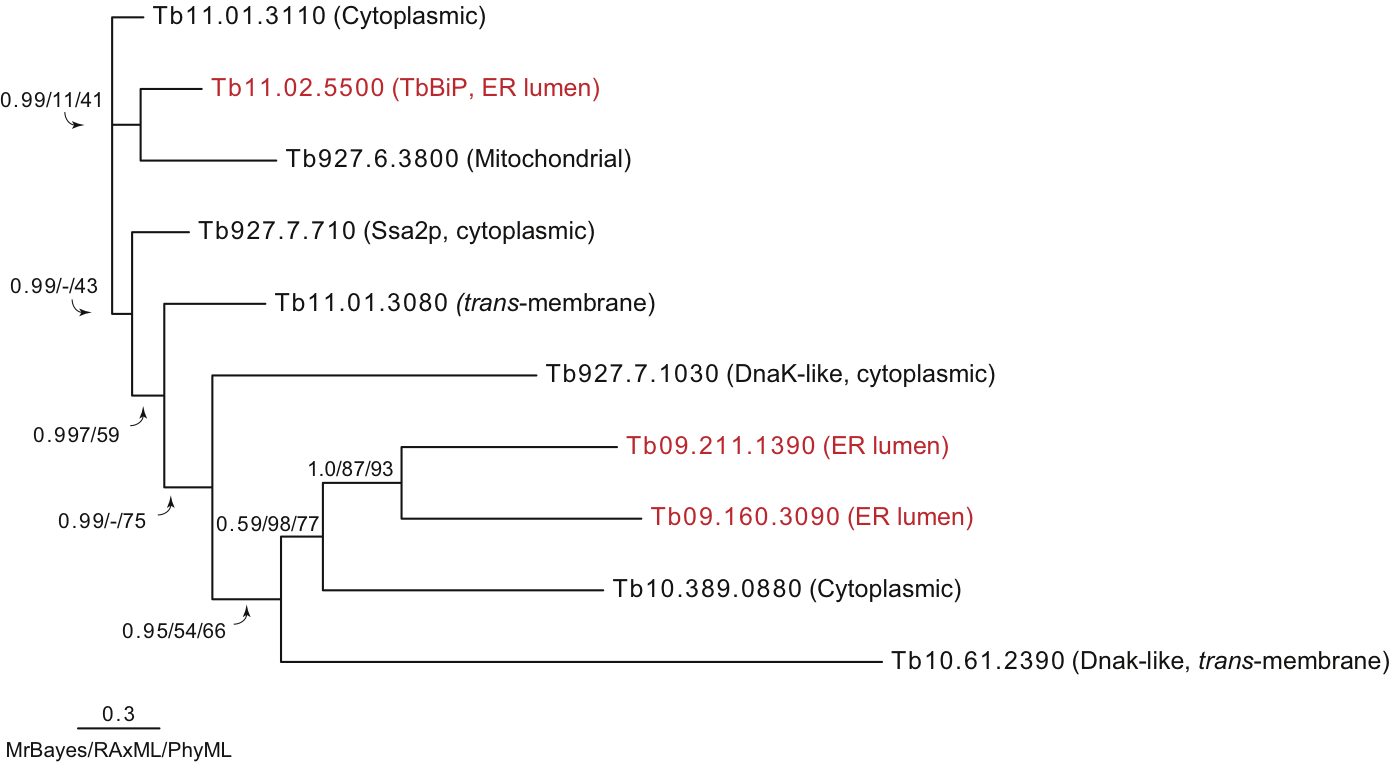

Supplement: Figure S2 — Phylogenetic reconstruction of part of the trypanosome Hsp70 family. Sequences for representative members of the trypanosome Hsp70 family were retrieved from geneDB. Sequences were aligned in Clustal, manually edited in MacClade and subjected to phylogenetic analysis. Gene products in red were analysed. Values at the internodes are bootstrap/bootstrap/posterior probability for RaXML, PhyML and Mr Bayes reconstructions. Annotations based on BLAST similarity to sequences at NCBI nr database and PSORT II are also provided. Note that most of these annotations should be considered tentative. (3.19 MB TIF) [file pone.0008468.s002.tif]

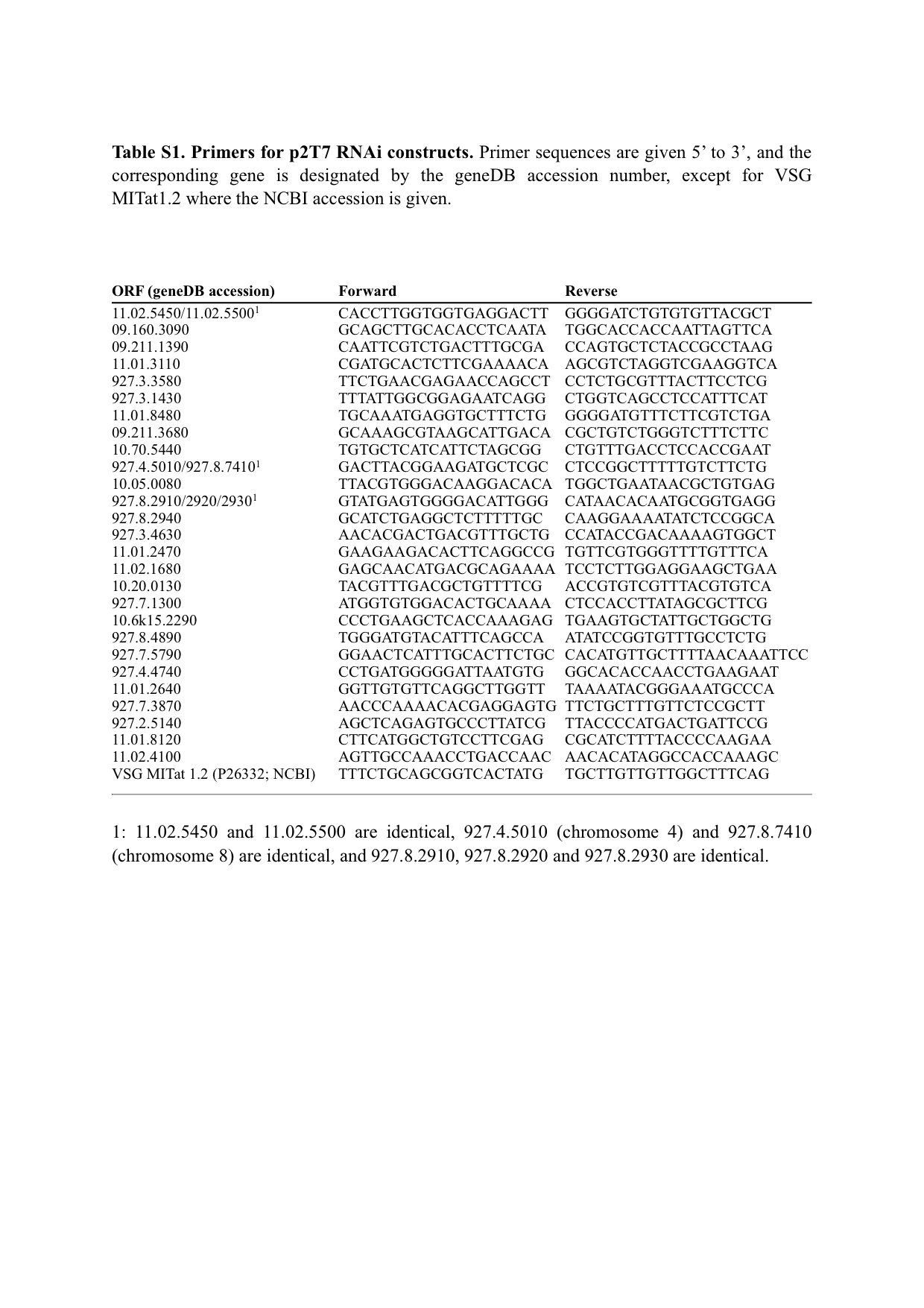

Supplement: Table S1 — Primers for p2T7 RNAi constructs. Primer sequences are given 5′ to 3′, and the corresponding gene is designated by the geneDB accession number, except for VSG MITat1.2 where the NCBI accession is given. (6.53 MB TIF) [file pone.0008468.s005.tif]

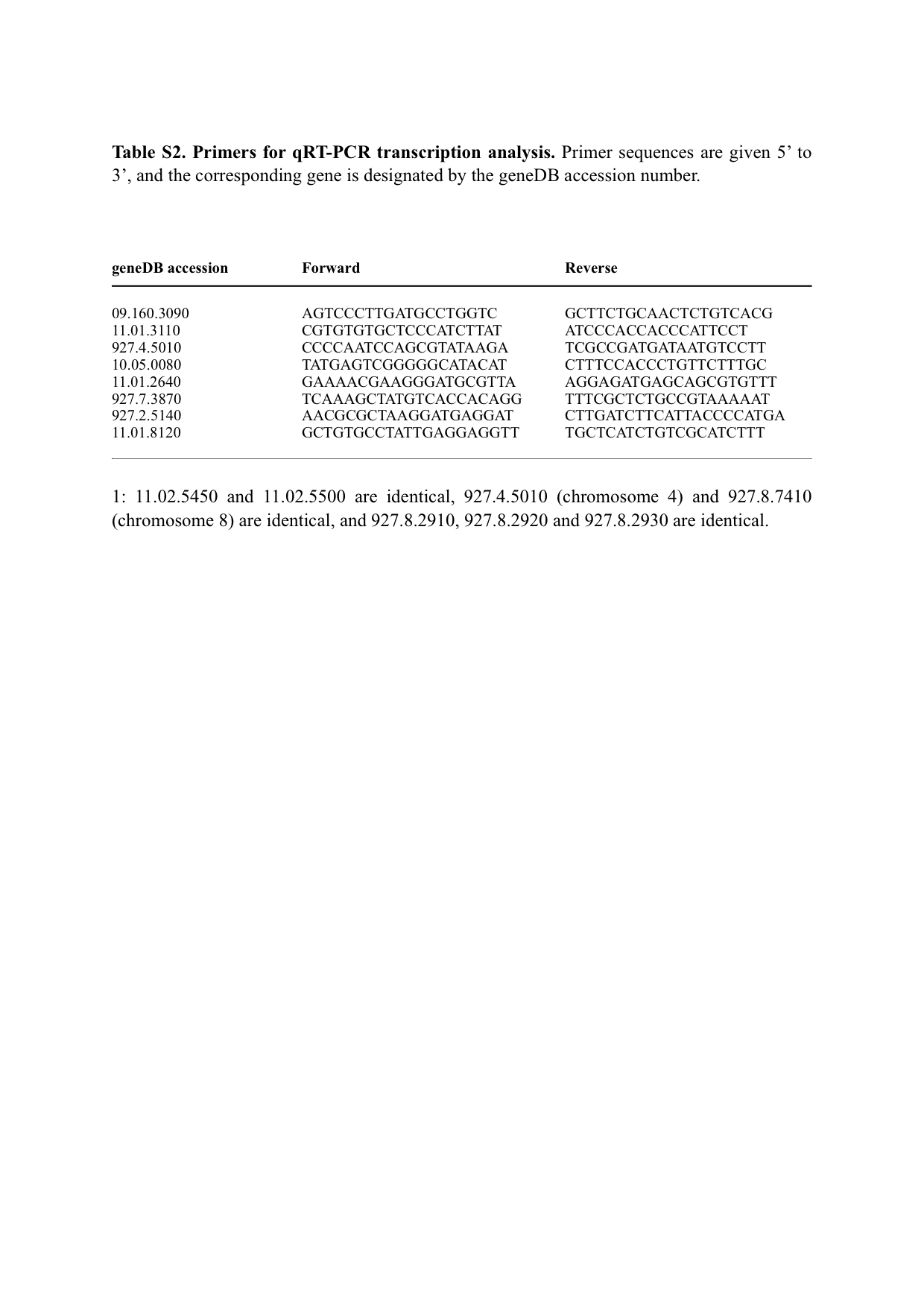

Supplement: Table S2 — Primers for qRT-PCR transcription analysis. Primer sequences are given 5′ to 3′, and the corresponding gene is designated by the geneDB accession number. (6.53 MB TIF) [file pone.0008468.s006.tif]
