# Supplementary material for: Chaperone Requirements for Biosynthesis of the Trypanosome Variant Surface Glycoprotein
Source: PLoS One. 2010 Jan 5;5(1):e8468. doi: 10.1371/journal.pone.0008468 (PMC2797082; doi:10.1371/journal.pone.0008468)
Supplement: Figure S4 — Clustal aligmnents for predicted amino acid and DNA sequences of EDEM ORFs from T. brucei. Sequences corresponding to geneDB accessions Tb927.8.2910, Tb927.8.2920, Tb927.8.2930 and Tb927.8.2940 were aligned with Clustal X using default parameters. (0.06 MB DOC) [file pone.0008468.s004.doc]

Predicted amino acid

Tb927.8.2940 MKGAQFLKMPCVRVLLVLVRVFFVHLPSFAFGDAFPVNGARGGNSQGYNTDGMHPIQAEM

Tb927.8.2930 MKGAQFLKMPCVRVLLVLVRVFFVHLPSFAFGDAFPVNGARGGNSQGYNTDGMHPIQAEM

Tb927.8.2920 MKGAQFLKMPCVRVLLVLVRVFFVHLPSFAFGDAFPVNGARGGNSQGYNTDGMHPIQAEM

Tb927.8.2910 --------MPWVRALLVLVTVFFVRLPSVMREERFHQYGN-GESRKLHNVSDMHPIQAEM

** **.***** ****:***. : * * * . : :*...********

Tb927.8.2940 LPYVRDMIDHAFGSYIKYAFPKDELCPVSGTGKNTMGGYGWTLIDSLDTLAIAGFHKEFR

Tb927.8.2930 LPYVRDMIDHAFGSYIKYAFPKDELCPVSGTGKNTMGGYGWTLIDSLDTLAIAGFHKEFR

Tb927.8.2920 LPYVRDMIDHAFGSYIKYAFPKDELCPVSGTGKNTMGGYGWTLIDSLDTLAIAGFHKEFR

Tb927.8.2910 LPYVRDMIDHAFGSYIKYAFPKDELCPVSGTGKNTMGGYGWTLIDSLDTLAIAGFHKEFR

************************************************************

Tb927.8.2940 RHAKWVEEHLTFDIDESVSVFETTIRALGGLLAAHFMYEEGIVPIIPSEHDYNGGFLRLA

Tb927.8.2930 RHAKWVEEHLTFDIDESVSVFETTIRALGGLLAAHFMYEEGIVPIIPSEHDYNGGFLRLA

Tb927.8.2920 RHAKWVEEHLTFDIDESVSVFETTIRALGGLLAAHFMYEEGIVPIIPSEHDYNGGFLRLA

Tb927.8.2910 RHAKWVEEHLTFDIDESVSVFETTIRALGGLLAAHFMYEEGIVPIIPSEHDYNGGFLRLA

************************************************************

Tb927.8.2940 VDLADRLMPCFDTPTGIPYGKVNLRSKSKEMWISRSNTAEVGTMLMEMTVLSRITGDEKY

Tb927.8.2930 VDLADRLMPCFDTPTGIPYGKVNLRSKSKEMWISRSNTAEVGTMLMEMTVLSRITGDEKY

Tb927.8.2920 VDLADRLMPCFDTPTGIPYGKVNLRRGVSGGESQLANTAGSGTLLVEMTVLSRITGDGKY

Tb927.8.2910 VDLADRLMPCFDTPTGIPYGAINLRRGVSGGESQLANTAGAGTLLMEMTVLSRITGDEKY

******************** :*** . . :*** **:*:*********** **

Tb927.8.2940 ERAARRASEALFAARDSQTELMGTYVSVSSGGFSSSESSVAGNIDSVIEYFIKSHSMSGD

Tb927.8.2930 ERAARRASEALFAARDSQTELMGTYVSVSSGGFSSSESSVAGNIDSAIEYFIKSHSMSGD

Tb927.8.2920 ERAARRASEALFAARDSQTELMGTYVSVSSGGFSSSESSVGSGLDSAIEYFIKSHSMSGD

Tb927.8.2910 ERAARRASEALFAARDSQTELMGNHIHTHTGIWRHGESSVGGNIDSVIEYFIKSHSMSGD

***********************.:: . :* : .****...:**.*************

Tb927.8.2940 IGDWERFERTARAVNRYVRKGGMLLAASMYSGRRLQTSQESLSSFFPGNLVLGGHLHEAV

Tb927.8.2930 IGDWERFERTARAVNRYVRKGGMLLAASMYSGRRLQTSQESLSSFFPGNLVLGGHLHEAV

Tb927.8.2920 IGDWERFERTARAVNRYVRKGGMLLAASMYSGRRLQTSQESLSSFFPGNLVLGGHLHEAV

Tb927.8.2910 IGDWERFERTARAVNRYVRKGGMLLAASMYSGRRLQTSQESLSSFFPGNLVLGGHLHEAV

************************************************************

Tb927.8.2940 ESSWPIHTFFKHFGVLPEIFSLESGEPSWISHDYIGRPEHIESLYMLYRATRDPTYLLMG

Tb927.8.2930 ESSWPIHTFFKHFGVLPEIFSLESGEPSWISHDYIGRPEHIESLYMLYRATRDPTYLLMG

Tb927.8.2920 ESSWPIHTFFKHFGVLPEIFSLESGEPSWRSHDYIGRPEHIESLYMLYRATRDPTYLLMG

Tb927.8.2910 ESSWPIHTFFKHFGVLPEIFSLESGEPSWMSHDYVGRPEHIESLYMLYRATRDPTYLLMG

***************************** ****:*************************

Tb927.8.2940 KELALAINLPALSCFFF-------------------------------------------

Tb927.8.2930 KELALAINLRMRTPYGFSSVSDVRYPHHDGVHRDSMESFMIAETLKYLYLLFDECNAVHM

Tb927.8.2920 KELALAINLRMRTPYGFSSVSDVRYPHHDGVHRDSMESFMIAETLKYLYLLFDECNAVHV

Tb927.8.2910 KELALAINLRMRTPYGFSSVSDVRYPHHDGVHRDSMESFMIAETLKYLYLLFDECNAVHM

********* : : *

Tb927.8.2940 ------------------------------------------------------------

Tb927.8.2930 QGRMGGRASPHCVMDSGSGSSVSHVGWVFNTEAHLFPNSAEWWAPTSLETLDKEAEDPAA

Tb927.8.2920 QGRMGGRASPHCVMDSGSGSSVSHVGWVFNTEAHLFPNSAEWWAPTSLETLDKEAEDPAA

Tb927.8.2910 QGRMGGRASPHCVMDSGSGSSVSHVGWVFNTEAHLFPNSAEWWAPTSLETLDKEAEDPAA

Tb927.8.2940 ----------------------------------------------------------

Tb927.8.2930 ALRRQRLEVIDGLLASFEEVDGEVVGANDKGGAALYQFHCANHALSDVGRLSKSVFR-

Tb927.8.2920 ALRRQRLEVIDGLLASFEEVDGEVVGANDKGGAALYQFHCANHALSDVGRLSKSVFR-

Tb927.8.2910 ALRRQRLEVIDGLLASFEEVDGEVVGANDKGGAALYQFHCANHALSDIGRLSKSVFR-

DNA

Tb927.8.2930 ATGAAAGGCGCTCAGTTTTTGAAGATGCCGTGTGTGCGTGTTTTACTAGTTCTCGTGAGG

Tb927.8.2940 ATGAAAGGCGCTCAGTTTTTGAAGATGCCGTGTGTGCGTGTTTTACTAGTTCTCGTGAGG

Tb927.8.2920 ATGAAAGGCGCTCAGTTTTTGAAGATGCCGTGTGTGCGTGTTTTACTAGTTCTCGTGAGG

Tb927.8.2910 ------------------------ATGCCGTGGGTTCGTGCTTTACTAGTTCTCGTGACG

******** ** **** ***************** *

Tb927.8.2930 GTATTTTTTGTCCATCTGCCTTCTTTTGCTTTTGGGGATGCGTTTCCTGTGAATGGTGCG

Tb927.8.2940 GTATTTTTTGTCCATCTGCCTTCTTTTGCTTTTGGGGATGCGTTTCCTGTGAATGGTGCG

Tb927.8.2920 GTATTTTTTGTCCATCTGCCTTCTTTTGCTTTTGGGGATGCGTTTCCTGTGAATGGTGCG

Tb927.8.2910 GTATTTTTTGTCCGTCTTCCGTCCGTGATGAGAGAGGAAAGGTTCCACCAGTACGGAAAT

************* *** ** ** * * *** *** * * * **

Tb927.8.2930 CGAGGAGGTAACAGCCAGGGCTACAATACCGATGGCATGCATCCTATTCAAGCTGAGATG

Tb927.8.2940 CGAGGAGGTAACAGCCAGGGCTACAATACCGATGGCATGCATCCTATTCAAGCTGAGATG

Tb927.8.2920 CGAGGAGGTAACAGCCAGGGCTACAATACCGATGGCATGCATCCTATTCAAGCTGAGATG

Tb927.8.2910 GGTGAAAGTAGAA---AGTTGCATAATGTCAGCGATATGCATCCTATTCAAGCTGAGATG

* * * *** * ** * *** * * ************************

Tb927.8.2930 CTTCCTTATGTGCGTGACATGATTGACCACGCGTTTGGTTCATACATCAAATACGCCTTT

Tb927.8.2940 CTTCCTTATGTGCGTGACATGATTGACCACGCGTTTGGTTCATACATCAAATACGCCTTT

Tb927.8.2920 CTTCCTTATGTGCGTGACATGATTGACCACGCGTTTGGTTCATACATCAAATACGCCTTT

Tb927.8.2910 CTTCCTTATGTGCGTGACATGATTGACCACGCGTTTGGTTCATACATCAAATACGCCTTT

************************************************************

Tb927.8.2930 CCCAAAGATGAATTGTGTCCTGTGAGTGGTACTGGGAAGAATACGATGGGTGGCTATGGC

Tb927.8.2940 CCCAAAGATGAATTGTGTCCTGTGAGTGGTACTGGGAAGAATACGATGGGTGGCTATGGC

Tb927.8.2920 CCCAAAGATGAATTGTGTCCTGTGAGTGGTACTGGGAAGAATACGATGGGTGGCTATGGC

Tb927.8.2910 CCCAAAGATGAATTGTGTCCTGTGAGTGGTACTGGGAAGAATACGATGGGTGGCTATGGC

************************************************************

Tb927.8.2930 TGGACCCTTATTGACTCTCTCGATACACTAGCAATTGCCGGGTTTCACAAAGAATTTCGT

Tb927.8.2940 TGGACCCTTATTGACTCTCTCGATACACTAGCAATTGCCGGGTTTCACAAAGAATTTCGT

Tb927.8.2920 TGGACCCTTATTGACTCTCTCGATACACTAGCAATTGCCGGGTTTCACAAAGAATTTCGT

Tb927.8.2910 TGGACCCTTATTGACTCTCTCGATACACTAGCAATTGCCGGGTTTCACAAAGAATTTCGT

************************************************************

Tb927.8.2930 CGCCACGCGAAGTGGGTGGAAGAGCACTTGACCTTCGATATTGACGAATCAGTGTCGGTA

Tb927.8.2940 CGCCACGCGAAGTGGGTGGAAGAGCACTTGACCTTCGATATTGACGAATCAGTGTCGGTA

Tb927.8.2920 CGCCACGCGAAGTGGGTGGAAGAGCACTTGACCTTCGATATTGACGAATCAGTGTCGGTA

Tb927.8.2910 CGCCACGCGAAGTGGGTGGAAGAGCACTTGACCTTCGATATTGACGAATCAGTGTCGGTA

************************************************************

Tb927.8.2930 TTTGAGACGACTATTCGAGCTCTTGGAGGTCTTCTGGCTGCTCACTTCATGTACGAGGAG

Tb927.8.2940 TTTGAGACGACTATTCGAGCTCTTGGAGGTCTTCTGGCTGCTCACTTCATGTACGAGGAG

Tb927.8.2920 TTTGAGACGACTATTCGAGCTCTTGGAGGTCTTCTGGCTGCTCACTTCATGTACGAGGAG

Tb927.8.2910 TTTGAGACGACTATTCGAGCTCTTGGAGGTCTTCTGGCTGCTCACTTCATGTACGAGGAG

************************************************************

Tb927.8.2930 GGCATAGTCCCAATTATCCCTTCGGAGCACGACTATAACGGCGGGTTCTTGCGGCTCGCT

Tb927.8.2940 GGCATAGTCCCAATTATCCCTTCGGAGCACGACTACAACGGCGGGTTCTTGCGGCTCGCT

Tb927.8.2920 GGCATAGTCCCAATTATCCCTTCGGAGCACGACTACAACGGCGGGTTCTTGCGGCTCGCT

Tb927.8.2910 GGCATAGTCCCAATTATCCCTTCGGAGCACGACTACAACGGCGGGTTCTTGCGGCTCGCT

*********************************** ************************

Tb927.8.2930 GTGGATCTTGCAGATCGTCTGATGCCCTGTTTTGACACGCCCACTGGGATACCATATGGA

Tb927.8.2940 GTGGATCTTGCAGATCGTCTGATGCCCTGTTTTGACACGCCCACTGGGATACCATATGGA

Tb927.8.2920 GTGGATCTTGCAGATCGTCTGATGCCCTGTTTTGACACGCCCACTGGGATACCATATGGA

Tb927.8.2910 GTGGATCTTGCAGATCGTCTGATGCCCTGTTTTGACACGCCCACTGGGATACCATATGGG

***********************************************************

Tb927.8.2930 AAGGTTAATTTACGCAGTAAAAGTAAGGAAATGTGGATTTCGAGATCAAATACAGCTGAA

Tb927.8.2940 AAGGTTAATTTACGCAGTAAAAGTAAGGAAATGTGGATTTCGAGATCAAATACAGCTGAA

Tb927.8.2920 AAGGTTAATTTACGCCGTGGGGTTAGTGGTGGGGAATCGCAGCTGGCCAACACGGCCGGT

Tb927.8.2910 GCGATTAATTTACGCCGTGGGGTTAGTGGTGGGGAATCGCAGCTGGCCAACACGGCCGGC

* *********** ** ** * * * * ** ** ** *

Tb927.8.2930 GTTGGCACGATGTTAATGGAAATGACGGTACTGTCGAGGATCACAGGCGATGAAAAATAC

Tb927.8.2940 GTTGGCACGATGTTAATGGAAATGACGGTACTGTCGAGGATCACAGGCGATGAAAAATAC

Tb927.8.2920 TCTGGGACGTTGTTAGTGGAGATGACGGTACTGTCGAGGATCACAGGCGATGGAAAATAC

Tb927.8.2910 GCTGGGACGTTGTTAATGGAGATGACGGTACTGTCGAGGATCACAGGCGATGAAAAATAC

*** *** ***** **** ******************************* *******

Tb927.8.2930 GAGCGTGCGGCGCGGCGTGCATCTGAGGCTCTTTTTGCGGCCAGAGATTCTCAAACTGAG

Tb927.8.2940 GAGCGTGCGGCGCGGCGTGCATCTGAGGCTCTTTTTGCGGCCAGAGATTCTCAAACTGAG

Tb927.8.2920 GAGCGTGCGGCGCGGCGTGCATCTGAGGCTCTTTTTGCGGCCAGAGATTCTCAAACTGAG

Tb927.8.2910 GAGCGTGCGGCGCGGCGTGCATCTGAGGCTCTTTTTGCGGCCAGAGATTCTCAAACTGAG

************************************************************

Tb927.8.2930 CTTATGGGGACGTATGTGTCTGTGAGTAGTGGCGGTTTTTCCTCTTCGGAGTCTTCTGTT

Tb927.8.2940 CTTATGGGGACGTATGTGTCTGTGAGTAGTGGCGGTTTTTCCTCTTCGGAGTCTTCTGTT

Tb927.8.2920 CTTATGGGGACGTATGTGTCTGTGAGTAGTGGCGGTTTTTCCTCTTCGGAGTCTTCTGTG

Tb927.8.2910 CTTATGGGGAATCACATTCATACTCATACAGGAATTTGGAGACATGGTGAATCATCCGTT

********** * * * ** ** ** * ** ** ** **

Tb927.8.2930 GCAGGGAACATTGATAGCGCCATTGAATACTTTATCAAATCACATAGTATGAGTGGGGAC

Tb927.8.2940 GCAGGGAACATTGATAGCGTCATTGAATACTTTATCAAATCACATAGTATGAGTGGGGAC

Tb927.8.2920 GGTTCTGGATTAGACAGCGCCATTGAATACTTTATCAAATCACATAGTATGAGTGGGGAC

Tb927.8.2910 GGAGGGAACATTGATAGCGTCATTGAATACTTTATCAAATCACATAGTATGAGTGGGGAC

* * ** **** ****************************************

Tb927.8.2930 ATTGGGGACTGGGAGCGGTTCGAGAGGACTGCGAGAGCTGTGAACCGCTATGTGCGGAAG

Tb927.8.2940 ATTGGGGACTGGGAGCGGTTCGAGAGGACTGCGAGAGCTGTGAACCGCTATGTGCGGAAG

Tb927.8.2920 ATTGGGGACTGGGAGCGGTTCGAGAGGACTGCGAGAGCTGTGAACCGCTATGTGCGGAAG

Tb927.8.2910 ATTGGGGACTGGGAGCGGTTCGAGAGGACTGCGAGAGCTGTGAACCGCTATGTGCGGAAG

************************************************************

Tb927.8.2930 GGTGGAATGCTGCTAGCAGCTAGCATGTATAGTGGACGGCGGCTGCAGACGTCCCAGGAA

Tb927.8.2940 GGTGGAATGCTGCTAGCAGCTAGCATGTATAGTGGACGGCGGCTGCAGACGTCCCAGGAA

Tb927.8.2920 GGTGGAATGCTGCTAGCAGCTAGCATGTATAGTGGACGGCGGCTGCAGACGTCCCAGGAA

Tb927.8.2910 GGTGGAATGCTGCTAGCAGCTAGCATGTATAGTGGACGGCGGCTGCAGACGTCCCAGGAA

************************************************************

Tb927.8.2930 TCTTTGTCATCATTTTTTCCTGGGAACTTGGTTCTTGGCGGCCATCTCCACGAGGCTGTA

Tb927.8.2940 TCTTTGTCATCATTTTTTCCTGGGAACTTGGTTCTTGGCGGCCATCTCCACGAGGCTGTA

Tb927.8.2920 TCTTTGTCATCATTTTTTCCTGGGAACTTGGTTCTTGGCGGCCATCTCCACGAGGCTGTA

Tb927.8.2910 TCTTTGTCATCATTTTTTCCTGGGAACTTGGTTCTTGGCGGCCATCTCCACGAGGCTGTA

************************************************************

Tb927.8.2930 GAAAGTTCGTGGCCAATTCATACGTTCTTCAAACATTTTGGCGTTCTGCCGGAGATATTT

Tb927.8.2940 GAAAGTTCGTGGCCAATTCATACGTTCTTCAAACATTTTGGCGTTCTGCCGGAGATATTT

Tb927.8.2920 GAAAGTTCGTGGCCAATTCATACGTTCTTCAAACATTTTGGCGTTCTGCCGGAGATATTT

Tb927.8.2910 GAAAGTTCGTGGCCAATTCATACGTTCTTCAAACATTTTGGCGTTCTGCCGGAGATATTT

************************************************************

Tb927.8.2930 TCCTTGGAATCTGGCGAGCCGTCGTGGATATCACATGACTACATTGGTCGACCTGAGCAT

Tb927.8.2940 TCCTTGGAATCTGGCGAGCCGTCGTGGATATCACATGACTACATTGGTCGACCTGAGCAT

Tb927.8.2920 TCCTTGGAATCTGGCGAGCCGTCGTGGAGGTCACATGACTACATTGGTCGACCTGAGCAT

Tb927.8.2910 TCCTTGGAATCTGGCGAGCCGTCGTGGATGTCACATGACTACGTTGGTCGACCTGAGCAT

**************************** ************ *****************

Tb927.8.2930 ATCGAGTCTCTGTATATGCTTTACCGTGCAACACGAGATCCTACATACCTCTTGATGGGA

Tb927.8.2940 ATCGAGTCTCTGTATATGCTTTACCGTGCAACACGAGATCCTACATACCTCTTGATGGGA

Tb927.8.2920 ATCGAGTCTCTGTATATGCTTTACCGTGCAACACGAGATCCTACATACCTCTTGATGGGA

Tb927.8.2910 ATCGAGTCTCTGTATATGCTTTACCGTGCAACACGAGATCCTACATACCTCTTGATGGGA

************************************************************

Tb927.8.2930 AAAGAACTGGCACTGGCCATTAACTTGCGTATGCGCACACCATATGGGTTTTCTTCCGTA

Tb927.8.2940 AAAGAACTGGCACTGGCCATTAACTTGCCTGCGT-----TGAGCTGTTTTTTTTTTTGA-

Tb927.8.2920 AAAGAACTGGCACTGGCCATTAACTTGCGTATGCGCACACCATATGGGTTTTCTTCCGTA

Tb927.8.2910 AAAGAACTGGCACTGGCCATTAACTTGCGTATGCGCACGCCATATGGGTTTTCTTCCGTA

**************************** * * * ** **** ** *

Tb927.8.2930 AGTGATGTAAGGTACCCGCATCATGATGGTGTTCACAGAGACTCGATGGAGAGTTTCATG

Tb927.8.2940 ------------------------------------------------------------

Tb927.8.2920 AGTGATGTAAGGTACCCGCATCATGATGGTGTTCACAGAGACTCGATGGAGAGTTTCATG

Tb927.8.2910 AGTGATGTAAGGTACCCGCATCATGATGGTGTTCACAGAGACTCGATGGAGAGTTTCATG

Tb927.8.2930 ATCGCGGAGACCCTGAAATATTTGTATCTCTTGTTTGACGAGTGCAACGCTGTTCACATG

Tb927.8.2940 ------------------------------------------------------------

Tb927.8.2920 ATCGCGGAGACCCTGAAATATTTGTATCTCTTGTTTGACGAGTGCAACGCTGTTCACGTG

Tb927.8.2910 ATCGCGGAGACCCTGAAATATTTGTATCTCTTGTTTGACGAGTGCAACGCTGTTCACATG

Tb927.8.2930 CAGGGACGGATGGGCGGTCGCGCCTCACCGCATTGTGTTATGGACAGCGGTAGCGGCAGT

Tb927.8.2940 ------------------------------------------------------------

Tb927.8.2920 CAGGGACGGATGGGCGGTCGCGCCTCACCGCATTGTGTTATGGACAGCGGTAGCGGCAGT

Tb927.8.2910 CAGGGACGGATGGGCGGTCGCGCCTCACCGCATTGTGTTATGGACAGCGGTAGCGGCAGT

Tb927.8.2930 AGCGTCAGCCACGTGGGGTGGGTTTTCAACACTGAAGCTCATCTGTTCCCTAATTCTGCC

Tb927.8.2940 ------------------------------------------------------------

Tb927.8.2920 AGCGTCAGCCACGTGGGGTGGGTTTTCAACACTGAAGCTCATCTGTTTCCTAATTCTGCC

Tb927.8.2910 AGCGTCAGCCACGTGGGGTGGGTTTTCAACACTGAAGCTCATCTGTTCCCTAATTCTGCC

Tb927.8.2930 GAGTGGTGGGCGCCTACTTCCTTGGAGACCTTGGACAAGGAGGCTGAGGATCCCGCTGCT

Tb927.8.2940 ------------------------------------------------------------

Tb927.8.2920 GAGTGGTGGGCGCCTACTTCCTTGGAGACCTTGGACAAGGAGGCTGAGGATCCCGCTGCT

Tb927.8.2910 GAGTGGTGGGCGCCTACTTCCTTGGAGACCTTGGACAAGGAGGCTGAGGATCCCGCTGCT

Tb927.8.2930 GCGCTCAGGCGCCAGCGTTTGGAGGTAATTGACGGTTTGCTCGCGAGTTTTGAGGAAGTG

Tb927.8.2940 ------------------------------------------------------------

Tb927.8.2920 GCGCTCAGGCGCCAGCGTTTGGAGGTAATTGACGGTTTGCTCGCGAGTTTTGAGGAAGTG

Tb927.8.2910 GCGCTCAGGCGCCAGCGTTTGGAGGTAATTGACGGTTTGCTCGCGAGTTTTGAGGAAGTG

Tb927.8.2930 GACGGTGAGGTTGTTGGGGCGAATGATAAGGGTGGTGCAGCCCTGTACCAGTTCCACTGT

Tb927.8.2940 ------------------------------------------------------------

Tb927.8.2920 GACGGTGAGGTTGTTGGGGCGAATGATAAGGGTGGTGCAGCCCTGTACCAGTTCCACTGT

Tb927.8.2910 GACGGTGAGGTTGTTGGGGCGAATGATAAGGGTGGTGCAGCCCTGTACCAGTTCCACTGT

Tb927.8.2930 GCCAATCACGCCCTGAGTGATGTAGGGAGGCTGTCGAAGTCTGTGTTTCGATAA

Tb927.8.2940 ------------------------------------------------------

Tb927.8.2920 GCCAATCACGCCCTGAGTGATGTAGGGAGGCTGTCGAAGTCTGTGTTTCGATAA

Tb927.8.2910 GCCAATCACGCCCTGAGTGATATAGGGAGGCTGTCGAAGTCTGTGTTTCGATAA
